# Genomic Evidence of an Ancient East Asian Divergence Event in Wild *Saccharomyces cerevisiae*

**DOI:** 10.1093/gbe/evab001

**Published:** 2021-01-11

**Authors:** Devin P Bendixsen, Noah Gettle, Ciaran Gilchrist, Zebin Zhang, Rike Stelkens

**Affiliations:** Division of Population Genetics, Department of Zoology, Stockholm University, Sweden

**Keywords:** *Saccharomyces cerevisiae*, yeast, long-read, genome assembly, structural variation, *Ty* element

## Abstract

Comparative genome analyses have suggested East Asia to be the cradle of the domesticated microbe Brewer’s yeast (*Saccharomyces cerevisiae*), used in the food and biotechnology industry worldwide. Here, we provide seven new, high-quality long-read genomes of nondomesticated yeast strains isolated from primeval forests and other natural environments in China and Taiwan. In a comprehensive analysis of our new genome assemblies, along with other long-read *Saccharomycetes* genomes available, we show that the newly sequenced East Asian strains are among the closest living relatives of the ancestors of the global diversity of Brewer’s yeast, confirming predictions made from short-read genomic data. Three of these strains (termed the East Asian Clade IX Complex here) share a recent ancestry and evolutionary history suggesting an early divergence from other *S. cerevisiae* strains before the larger radiation of the species, and prior to its domestication. Our genomic analyses reveal that the wild East Asian strains contain elevated levels of structural variations. The new genomic resources provided here contribute to our understanding of the natural diversity of *S. cerevisiae*, expand the intraspecific genetic variation found in this heavily domesticated microbe, and provide a foundation for understanding its origin and global colonization history.

**Significance**

Brewer’s yeast (*Saccharomyces cerevisiae*) is a domesticated microbe and research model organism with a global distribution, and suspected origin in East Asia. So far, only limited genomic resources are available from nondomesticated lineages. This study provides seven new, high-quality long-read genomes of strains isolated from primeval forests and other natural environments in China and Taiwan. Comparative genomics reveal elevated levels of structural variation in this group and early phylogenetic branching prior to the global radiation of the species. These new genomic resources expand our understanding of the evolutionary history of Brewer’s yeast and illustrate what the ancestors of this highly successful microbe may have looked like.

## Introduction

The history of Brewer’s yeast, *Saccharomyces cerevisiae*, is deeply interwoven with that of humanity, having played significant roles in cultural, technological, and societal development for at least 9,000 years (McGovern et al. 2004). Although over a hundred years of *S. cerevisiae* research has provided important insights into eukaryotic genomics, evolution and cell physiology, much of its “wild” ecology as well as its deep human and prehuman evolutionary history have, until recently, largely remained a mystery. Recent broadscale genomic surveys of *S. cerevisiae* and its close relatives, however, are beginning to shed light on important aspects of its population genetic structure, intra- and interspecific hybridization events, and their interplay in yeast domestication (Scannell et al. 2011; Wang et al. 2012; Duan et al. 2018; Peter et al. 2018).

One of the key results from these broadscale genomic surveys has been increasing evidence for a singular and central radiation event of *S. cerevisiae* from Far East Asia (Wang et al. 2012; Duan et al. 2018; Peter et al. 2018). These studies have independently revealed that strains of wild yeast collected in parts of China and Taiwan contain much higher genomic diversity and show greater levels of divergence than all other strains of *S. cerevisiae*. The vast majority of these *S. cerevisiae* genomes, however, have been analyzed using short-read sequencing, resulting in a focus on single-nucleotide variants. Larger structural variations (SVs), such as inversions, deletions, and gene duplications, in addition to repetitive regions such as transposable elements (TE) and telomeres, have gone largely unresolved (Goodwin et al. 2016). However, in recent years, especially in yeast, interest in resolving and understanding SVs has increased (Jeffares et al. 2017; Yue et al. 2017). In addition to playing significant roles in yeast adaptation (Payen et al. 2014; Steenwyk and Rokas 2018; Zhang et al. 2020), these large structural features can provide increased phylogenetic resolution and key insights about lineage interactions and potential reproductive isolation (Hou et al. 2014). SVs have shown to be vital for evolutionary adaptation in many other taxa, supporting the role of inversions in adaptation and speciation, and in the evolution of disease (Merker et al. 2018; Wellenreuther et al. 2019).

In this study, we generated high-quality assemblies of seven of the highly divergent wild East Asian strains and one common laboratory strain (table 1) using both short reads and PacBio long reads to better understand the relationships of these strains to the global diversity of *S. cerevisiae*. Analyzing our assemblies in the context of publicly available long-read genomes, we generated a new phylogeny that confirms the place of these East Asian strains at the base of *S. cerevisiae* and provides further evidence for an out-of-China colonization history of this species. Moreover, we were able to group our sequenced strains belonging to the previously identified CHN IX clade with a Taiwanese strain, both shown in separate studies to be divergent from the rest of *S. cerevisiae*. We show that this combined clade likely has deep roots in mainland China and has had little gene flow with other *S. cerevisiae* strains.

## Results

### Genome Sequencing and Assembly

We used whole-genome long-read PacBio sequencing to assemble the genomes of seven divergent and one common laboratory strain of *S. cerevisiae* (supplementary fig. S1, Supplementary Material online, average per base genomic coverage = 91.3; average median read length = 3,028 bp). Initial nuclear and mitochondrial assemblies were highly complete (median number of contigs = 25; median N50 = 821,424.5). Final nuclear and mitochondrial assemblies were further resolved to single contigs for each chromosome (supplementary table S1, Supplementary Material online, median N50 = 907,965.5). Final genome sizes ranged from 11.65 to 11.92 Mb. Assessment of the completeness of the genome assembly and annotation using BUSCO found that all genomes had similarly high BUSCO scores (*C* > 96.5%, supplementary table S2, Supplementary Material online).

### Phylogenomics

**Fig. 1:**
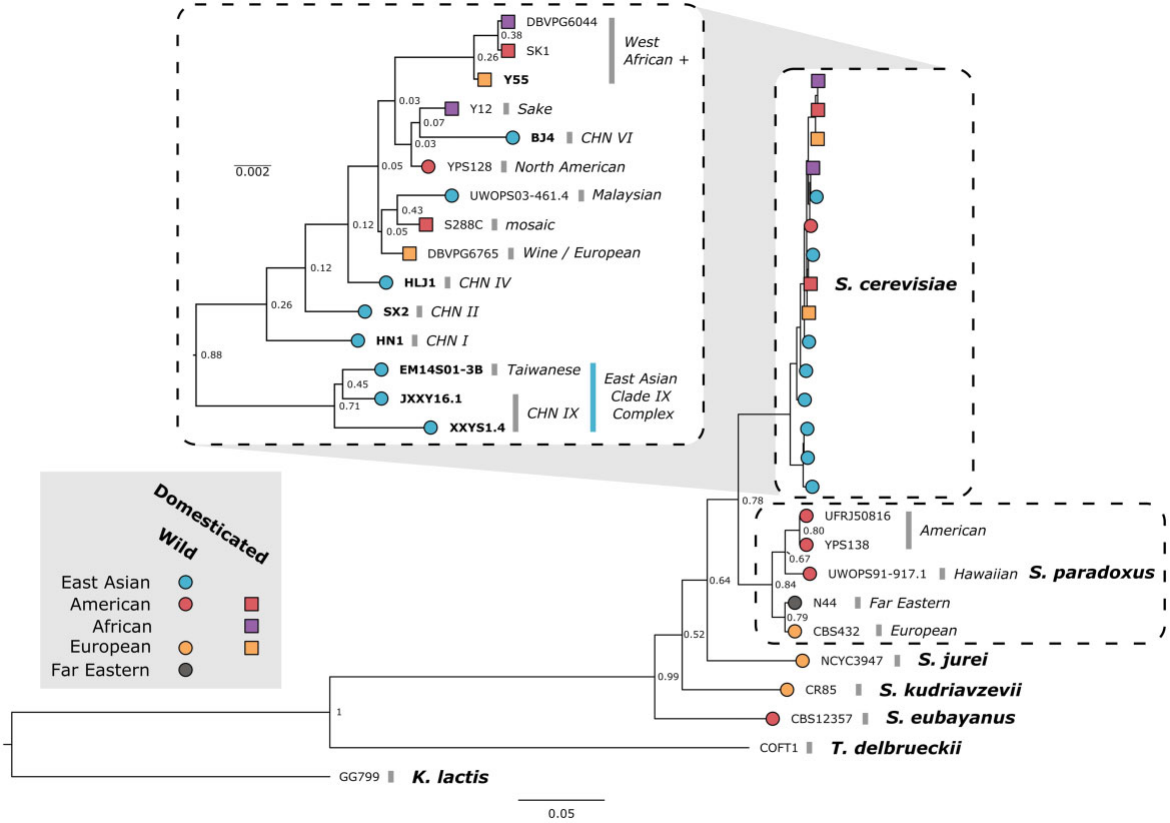
Consensus phylogenetic tree of yeast long-read genomes. The tree was built by orthogroup inference. The support values are the proportion of times that the bipartition is seen in each of the individual species tree estimates. Branch lengths represent the average number of substitutions per site across the sampled gene families. For species with more than a single long-read genome assembly (*Saccharomyces cerevisiae* and *Saccharomyces paradoxus*), species clades are indicated in italics. Strains are colored according to their location of origin and branch tip shape indicates whether it is domesticated (square) or wild (circle). Inset depicts *S. cerevisiae* strains with independent scaling. New long-read genome assemblies presented in this study are indicated in bold.

Our newly constructed consensus species tree placed six of the newly assembled East Asian strains in a basal position within the *S. cerevisiae* radiation (fig. 1). Three of these strains, EM14S01-3B (Taiwanese) (Peter et al. 2018), XXYS1.4, and JXXY16.1 (CHN IX) (Duan et al. 2018), hereon referred to as the East Asian Clade IX Complex, show early divergence from all other *S. cerevisiae* strains. Despite the largely basal placement of our assembled East Asian strains, one strain (BJ4) clustered separately with Y12 and YPS128, strains isolated from Ivory Coast palm wine and Pennsylvanian woodland soil, respectively. The common laboratory strain, Y55, clustered with two other domesticated strains (DBVPG6044 and SK1) within the West African+ clade. Construction of an Alignment and Assembly-Free (AAF) phylogeny comparing the long-read sequencing data generated in this study and previous short-read data found a high level of similarity between the two data sets (supplementary figs. S2 and S3, Supplementary Material online). This analysis also found similar clustering to the consensus species tree, among the East Asian strains and the common laboratory strain, as well as a large amount of divergence of the East Asian Clade IX Complex from the rest of *S. cerevisiae.* Furthermore, the removal of highly divergent genes (representing potential regions of introgression) unique to the Clade IX Complex did not significantly affect the phylogenetic topology or distance of this group relative to the rest of *S. cerevisiae* (supplementary fig. S4, Supplementary Material online). AAF was unable to resolve the early divergence of the East Asian Clade IX Complex from the rest of the species.

### Structural Variation

**Fig. 2:**
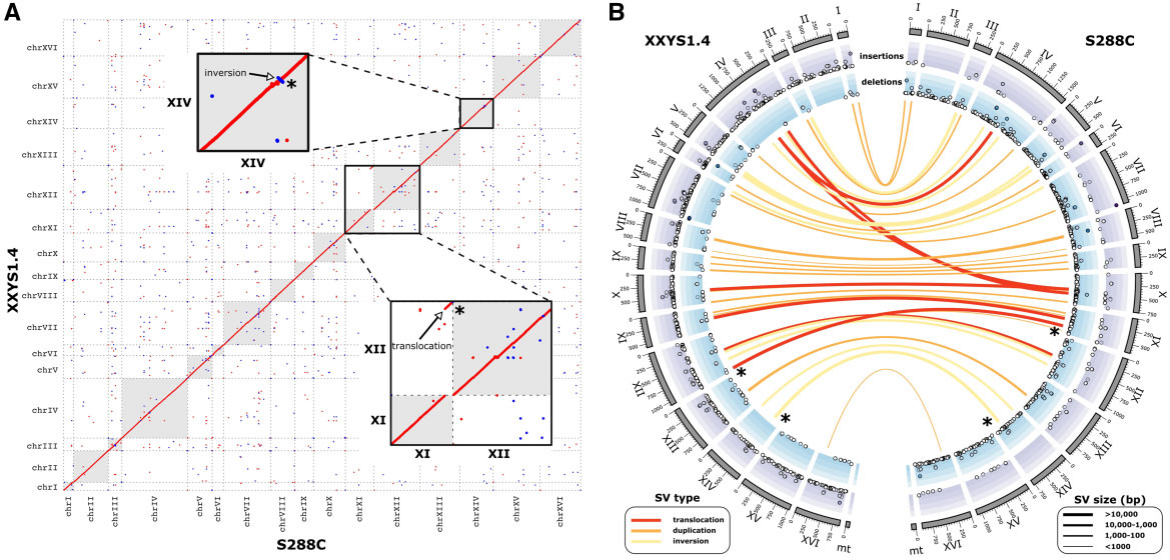
*Saccharomyces cerevisiae* long-read PacBio genome assemblies. (*A*) Genome comparison of the reference strain, S288C, and a member of the East Asian Clade IX Complex: XXYS1.4. Sequence homology within the dot plots is indicated by red dots for forward matches and blue dots for reverse matches. Insets depict examples of deviations from homology: 1) a large translocation between XI and chromosome XII found conserved in JXXY16.1, XXYS1.4, and EM14SO1-3B and 2) a large inversion in chromosome XIV. (*B*) CIRCOS plot showing the detected structural variations between reference strain, S288C and XXYS1.4. Translocations (red), duplications (orange), and inversions (yellow) are depicted as links between the two genomes. The width of the link reflects the relative size of the variation (bp). The translocation and inversion depicted in panel (*A*) are highlighted with asterisks. Insertions (blue) and deletions (purple) are depicted in the outer tracks. Deletion and insertion size increase toward the outside. Chromosome size is shown on the outside in 1-kb units.

A comparison of our eight *S. cerevisiae* genomes and previously assembled *Saccharomyces* sensu stricto genomes to the *S. cerevisiae* reference genome (S288C) revealed a high level of collinearity, particularly at larger scales (fig. 2 and supplementary figs. S5–S11, Supplementary Material online). We found exceptions to this strict collinearity only in one strain of *Saccharomyces paradoxus* (previously reported in Yue et al. 2017) and in the East Asian Clade IX Complex. All three member strains show an approximately 80-kb terminal translocation from chromosome XI to chromosome XII (fig. 2*A* inset). This structural variant in the East Asian Clade IX Complex was further supported by both long- and short-read analyses of alignment coverage (supplementary figs. S12 and S13, Supplementary Material online). Additional evidence for this unique translocation comes from high short-read coverage of chromosome XII of XXYS1.4 indicating a likely aneuploidy, which extends across the translocated region of chromosome XI (supplementary fig. S13, Supplementary Material online). Other notable rearrangements are a large inversion in chromosome X of BJ4 (supplementary fig. S6, Supplementary Material online). The common laboratory strain, Y55, showed a high level of collinearity with only minor deviations (supplementary fig. S5, Supplementary Material online).

**Fig. 3:**
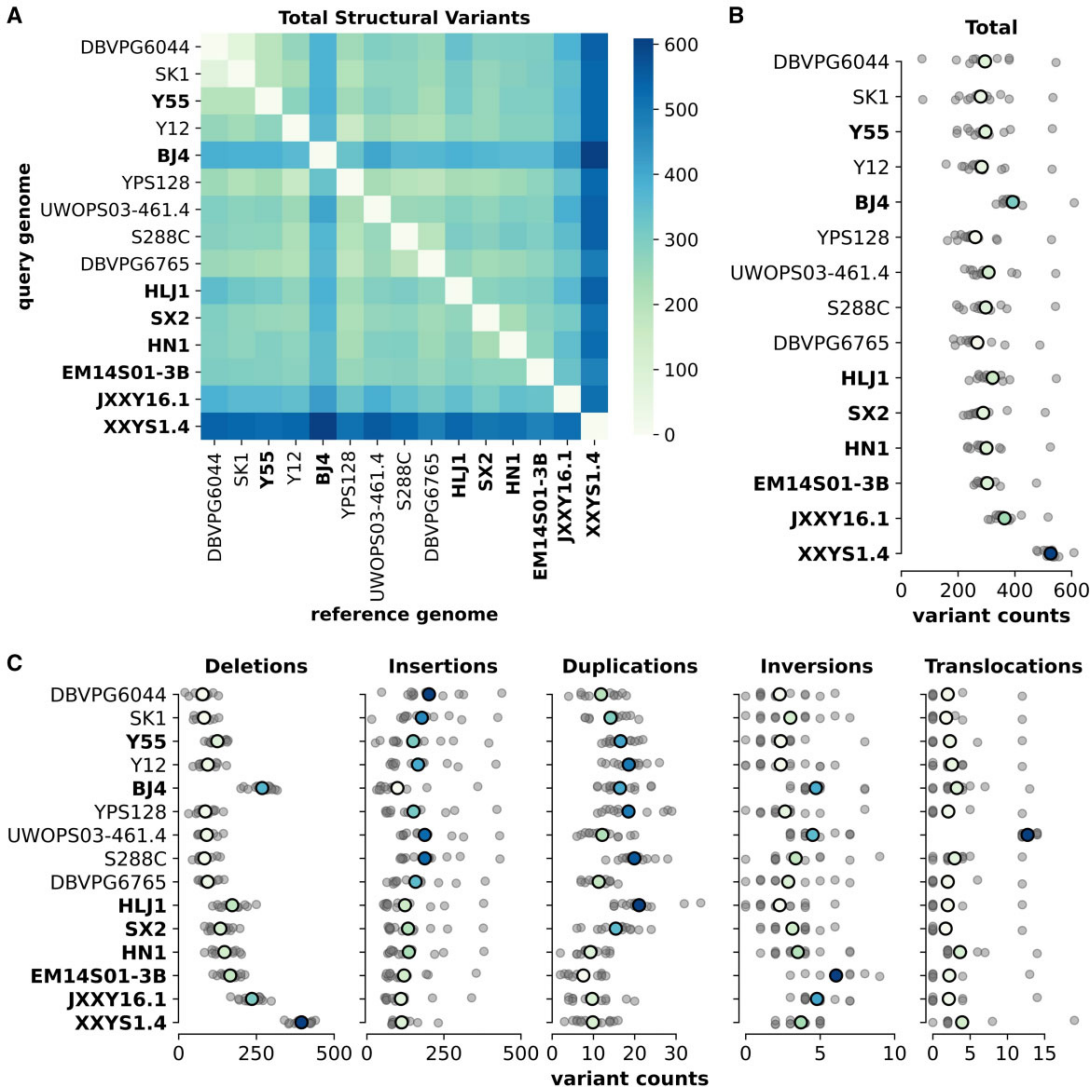
Structural variations within *Saccharomyces cerevisiae*. (*A*) Pairwise comparisons among all *S. cerevisiae* genome assemblies with the total number of variations. Order of genome assemblies is consistent with the species tree (fig. 1). New long-read genome assemblies presented in this study are bold. (*B*) The range of total structural variation counts found for each genome serving as reference genome. Gray dots indicate each pairwise genome comparison. Colored dots indicate the mean and are colored on a relative scale. (*C*) The range of structural variation counts for each type of variation. Gray dots indicate each pairwise genome comparison. Colored dots indicate the mean and are colored on a relative scale. Corresponding heatmaps for pairwise comparison are shown in supplementary figures S14–S18, Supplementary Material online.

To quantify the extent of smaller SVs in our genomes, we performed a comprehensive analysis using pairwise comparisons between the 15 *S. cerevisiae* strains with long-read assemblies. We assessed five types of variation: deletions, insertions, duplications, inversions, and translocations. This analysis revealed that the wild East Asian strains tend to have higher amounts of total variation (mean = 356.5 structural variant count) compared with the other strains (mean = 384.7, fig. 3*A*). The three Clade IX Complex strains (EM14S01-3B, JXXY16.1, and XXYS1.4) were among the highest, and in particular strain XXYS1.4 had a significantly higher mean structural variant count (525.9, fig. 3*B*). In contrast, the laboratory strain Y55 had more moderate levels of total variation. The East Asian Clade IX Complex also had larger numbers of deletions and inversions, and fewer insertions and duplications (fig. 3*C* and supplementary figs. S14–S18, Supplementary Material online). The Malaysian strain UWOPS03-461.4 had significantly larger numbers of translocations compared with all strains, agreeing with previous analyses of the strain (Yue et al. 2017). A closer analysis of the distribution of all SVs identified along chromosomes revealed areas of elevated variation counts; however, we found no strong patterns (supplementary fig. S19, Supplementary Material online).

### Nuclear Genome Content

In general, our newly assembled long-read genomes were significantly smaller than the currently existing genomes (*t* = 2.36, df = 10.28, *P* = 0.039). This difference, however, is largely a result of reduced genome size in members of the East Asian Clade IX Complex (${\bar{\text{X}}}_{\text{CladeIX}}=11.72\text{Mbp; }{\bar{\text{X}}}_{\text{CladeIX}}=11.89\text{Mbp; }$*t* = 2.93, df = 5.25, *P* = 0.031). These size differences are due to decreases in genic material both in terms of counts (*t* = 6.12, df = 10.43, *P* < 0.001) and cumulative gene length (*t* = 7.35, df = 5.0, *P* < 0.001), and a relative reduction in noncoding DNA (*t* = 4.95, df = 6.74, *P* = 0.002) (supplementary fig. S20, Supplementary Material online). Interestingly, these relative reductions in genic material are correlated with increases in identified intronic material, a pattern that is carried throughout all *S. cerevisiae* strains analyzed here (*F* = 11.38; *r*^2^ = 0.43; *P* = 0.005).

Genome size in the strains of *S. cerevisiae* we analyzed is correlated with gene number (*R*^2^ = 0.53; *P* = 0.001), a trend that is largely driven by gene loss rather than gene gain or amount of noncoding DNA (*F* = 15.432; *P* = 0.002) (supplementary fig. S21, Supplementary Material online). This trend holds true for the members of the East Asian Clade IX Complex, which have both smaller genomes and lower than average numbers of exons than the average for *S. cerevisiae* (supplementary fig. S20, Supplementary Material online). We identified 258 gene losses in the East Asian Clade IX Complex (supplementary table S3, Supplementary Material online) that show enrichment in the number of known interactions (*P* < 0.001) but, in terms GO functionality, only the seripauperin/TIP1 family was significantly enriched (FDR = 0.00017). More importantly, the East Asian Clade IX Complex lacks 45 genes found within the core genome of all the strains analyzed in this study. These genes, however, show no significant GO enrichments or position correlations indicating why they were lost (supplementary table S4, Supplementary Material online).

### TE Composition

TEs replicate and deteriorate in a way that gives them an evolutionary history that can be unique with regard to their host genomes and can provide hints about past interactions between distinct lineages. To better understand historical relationships between different strains of *S. cerevisiae*, we annotated and analyzed all classes of known retrotransposon or *Ty* element in this species.

**Fig. 4:**
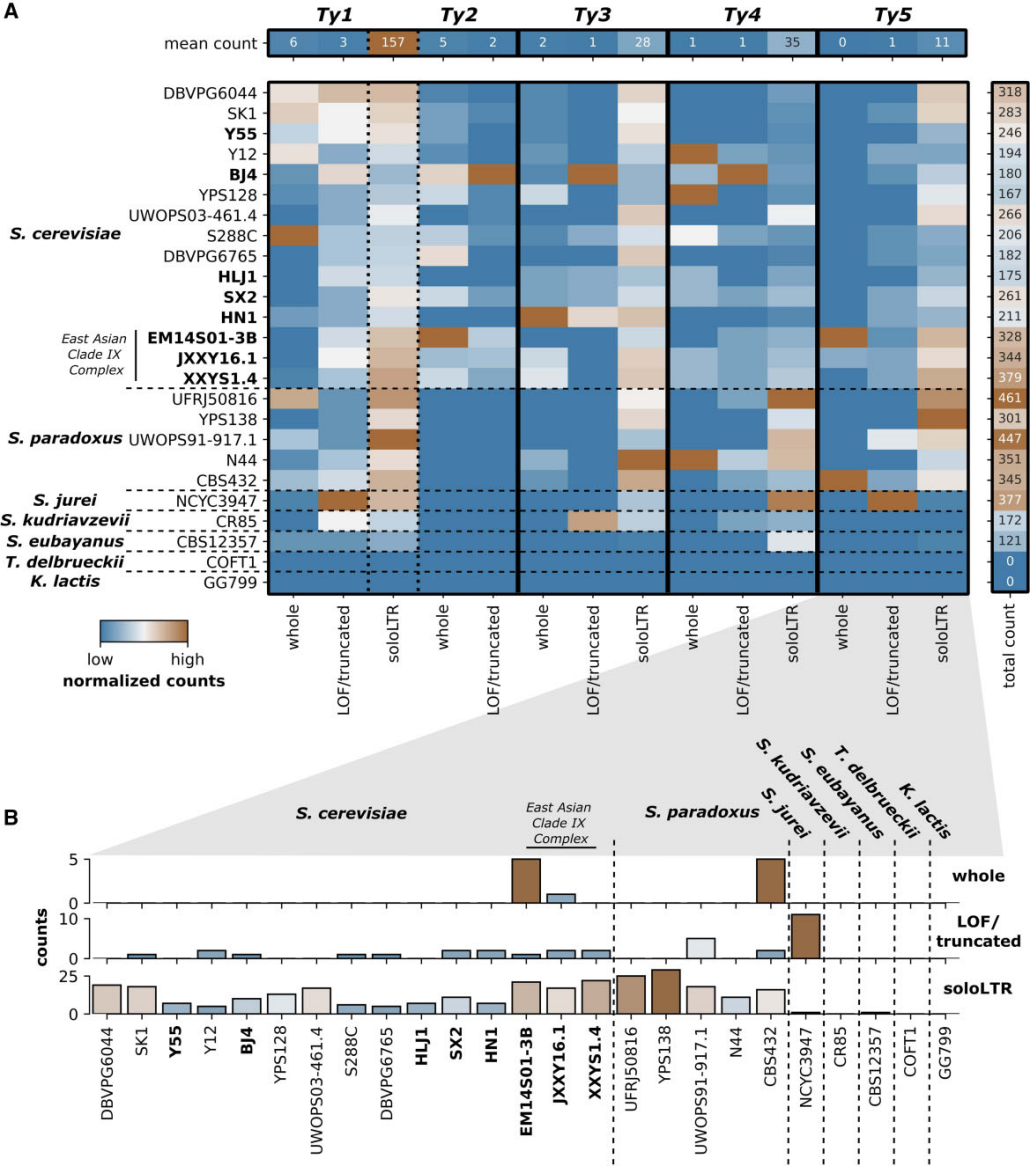
Transposable element composition in *Saccharomyces*. (*A*) Transposable element composition in total count subdivided by *Ty* classification for *Saccharomyces* sensu stricto strains. For visual comparison, each column is normalized (*x*/*x*_max_) for that specific element. For raw values, see supplementary figure S22, Supplementary Material online. LTR, long terminal repeat components of *Ty* elements without replicative machinery. (*B*) A closer look at *Ty5* elements across *Saccharomyces*.

In terms of simple counts, members of the East Asian Clade IX Complex had more *Ty*-associated elements than the rest of the *S. cerevisiae* strains (*t* = −6.05, df = 6.31, *P* < 0.001), a result largely based on a disproportionate number of solo long terminal repeats (LTRs) across all classes of *Ty* elements (fig. 4*A* and supplementary figs. S22 and S23, Supplementary Material online). A similar pattern remained when comparing total length of elements (supplementary fig. S23, Supplementary Material online). Although *Ty1*/*Ty2* LTRs were the most common *Ty* remnant in all strains, the relative frequency of each class of *Ty* element across *S. cerevisiae* strains does not follow the same pattern reported for the reference strain S288C, where *Ty1 *>* Ty2 *>* Ty3 *>* Ty4 *>* Ty5*. Indeed, *Ty1* elements have often been suggested as being the most prolific TE class in *S. cerevisiae*; however, we did not find any putatively functional *Ty1* elements in 6 of the 15 strains we analyze while finding 30 in the reference strain, S288C, representing a clear outlier at the upper end.

As yet, functional *Ty5* elements had only been identified in *S. paradoxus.* “Complete” elements (i.e., elements containing both flanking LTRs and the internal coding region) previously identified in *S. cerevisiae* strains are missing an approximately 2-kb portion of the approximately 5-kb internal coding region and are found in very low numbers (1–2 per strain). However, the Clade IX Complex strains show a particularly high abundance of *Ty5*-associated elements (fig. 4*B*). Further examination revealed six complete *Ty5* elements with fully intact coding regions distributed across two Clade IX Complex strains, EM14S01-3B and JXXY16 (supplementary fig. S25, Supplementary Material online). Although all “complete” *Ty5* elements that we identified in *S. cerevisiae* outside of the Clade IX Complex are missing the same approximately 2-kb region, only 2 out of 10 Clade IX *Ty5* elements (both in JXXY16.1) are missing this region. Additionally, these elements largely do not share homologous bordering regions. In conclusion, the only putatively functional *Ty5* elements in *S. cerevisiae* are in the Clade IX Complex.

### Comparative Mitochondrial Genomics

**Fig. 5:**
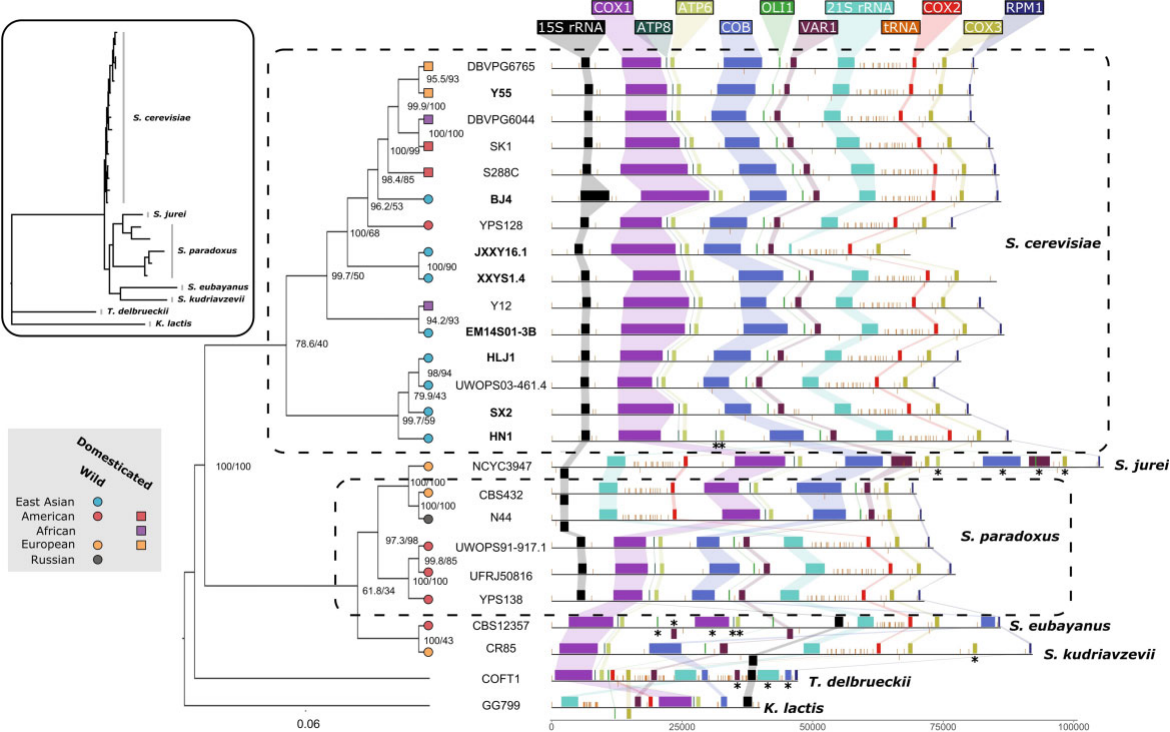
Mitochondrial phylogenetics and genomic arrangements. Phylogenetic tree based on mitochondrial genomic content. Internal branches are labeled with bootstrap support. *Saccharomyces* strains are colored according to their location of origin and branch tip shape indicates whether it is a domesticated (square) or wild (circle) strain. New long-read genome assemblies presented in this study are indicated in bold. Major genomic elements found on mitochondria are shown and colored according to guide elements at the top. Inverted elements appear on the underside of the line. Duplicated elements are indicated with asterisk. Inset depicts untransformed phylogenetic tree with species labeled.

Overall, the mitochondrial genomes of the *S. cerevisiae* strains show high levels of collinearity (fig. 5). Of note, however, is the absence of *RPM1*, a highly conserved ncRNA component of mitochondrial RNase P in two of the Clade IX Complex strains, JXXY16.1 and XXYS1.4. To further investigate the absence of this gene, we aligned the reference *RPM1* to the unassembled PacBio reads using BlastN (Zhang et al. 2000). We found no full-length alignments of *RPM1*, a 483-bp gene, in either set of reads; rather the highest scoring alignments (*e*-value > 9e-35) were 149 (JXXY16.1) and 239 bp (XXYS1.4) (supplementary fig. S26, Supplementary Material online). Some reads mapped to the reference sequence for longer lengths (∼300 bp), however, with lower alignment scores. We also performed the same analysis using the previously published short reads. Interestingly, all reads mapped to the same short region of the gene. Similarly, we were unable to assemble more than a truncated version of the mitochondrial *21s rRNA* in JXXY16.1. However, when analyzing the raw sequencing reads we found reads that mapped to portions of the gene (supplementary fig. S27, Supplementary Material online). Potential sequence divergence or truncation around the SCEI endonuclease might have caused poor alignment in the JXXY16.1 genome. None of the strains we sequenced was found to be respiratory incompetents or ρ–. This analysis suggests that *RPM1* and *21s rRNA* are likely found in these divergent yeast strains; however, truncation or sequence divergence limits their alignment and proper annotation.

Although the mitochondrial tree shows some degree of discordance with the species tree, particularly with respect to the position of the East Asian Clade IX Complex strains, we found that mitochondrial genes from this clade showed on average higher similarity to other *S. cerevisiae* mitochondrial genes (99.74–99.71% identity) than their nuclear counterparts (98.63–98.66% identity). Although mitochondrial introgression may have played a role in the evolution of these mitochondrial genomes, the degree of similarity and lack of genes make high resolution of the mitochondrial phylogeny difficult.

Previous analyses have suggested that hybridization events can generate discordance between species and mitochondrial phylogenies in yeast (Peris et al. 2017; De Chiara et al. 2020). To investigate this, we also included other *Saccharomyces* species with available long-read mitochondrial genomes. For the most part, our mitochondrial phylogenies matched our species-level phylogeny with the notable exception of a strain of *Saccharomyces jurei* (NCYC3947), a recently described European species (Naseeb et al. 2018) that appears to share mitochondrial ancestry with a subgroup of European strains of *S. paradoxus*. The mitochondrial genomes from this subgroup also contain large SVs (previously described in Yue et al. [2017]) not seen in other strains of *S. paradoxus* and *S. cerevisiae*, further supporting their shared ancestry (fig. 5).

### Intraspecific Spore Viability

We crossed each wild East Asian strain with the common laboratory strain Y55 to assess the level of reproductive isolation. As expected, we found a lower level of viable spores when crossing with a divergent wild strain as compared with self-crossing Y55 (ANOVA [analysis of variance] *F*(7,152) = 9.63, *P* < 0.001; supplementary fig. S28, Supplementary Material online). Most crosses with East Asian strains reduced spore viability by approximately 50%, whereas crosses with HN1 reduced viability by approximately 75%. To investigate the low viability of crosses with HN1, we also crossed HN1 to itself, which yielded 100% spore viability.

## Discussion

Comparative genomic analyses have provided clues about the origin of Brewer’s yeast and have suggested an out-of-China origin (Peter et al. 2018). Here, we provide seven new, high-quality long/short-read genomes of highly divergent wild *S. cerevisiae* strains recently isolated in Far East Asia. Phylogenomic analyses of the long-read assemblies agree with previous findings that the wild East Asian strains (CHN, Taiwanese) are basal relative to other *S. cerevisiae* strains (Duan et al. 2018; Peter et al. 2018) and, in the case of the CHN IX and Taiwanese clades, show considerable divergence (fig. 1). In addition, we show that the CHN IX clade (represented here by JXXY16.1 and XXYS1.4) and the strain representing the Taiwanese clade (EM14S01-3B) likely compose a single monophyletic group distinct from not only the other East Asian strains in our study but also all other strains of *S. cerevisiae* sequenced to date.

Our SV analysis further elucidates the evolutionary history and intraspecific diversity of *S. cerevisiae*. SVs identified for each strain pair revealed patterns of genomic divergence with higher amounts of SVs in wild East Asian strains, especially in the three strains within the Clade IX Complex. This is interesting because, as a species, *S. cerevisiae* has been shown to accumulate balanced variations at a slower rate compared with *S. paradoxus* (Yue et al. 2017). This is likely due to the different selection histories of these species; many *S. cerevisiae* strains have long been associated with human activities where domestication, cross-breeding, and admixture have resulted in largely mosaic genomes (Liti et al. 2009; Hyma and Fay 2013; Liti 2015), whereas *S. paradoxus* strains are recently isolated, wild strains. Interestingly, we found that wild East Asian strains accumulated both SVs at a high rate, more similar to rates normally seen in *S. paradoxus* (fig. 3). It has been suggested that the geographic isolation of some *S. paradoxus* subpopulations may have favored quick fixation of structural rearrangements (Leducq et al. 2016). We may be witnessing similar patterns in the wild East Asian *S. cerevisiae* strains.

Comparisons between our new long-read genomes and the seven previously assembled *S. cerevisiae* and other *Saccharomyces* species genomes reveal other important aspects of yeast evolutionary genomic history. Not only do the phylogenetic patterns we describe reveal discrete boundaries between certain clade levels in terms of TEs, indicating that transfer of persisting TEs between deep-rooted clades through either horizontal gene transfer or hybridization is rare (fig. 4), but they also give us context for the evolutionary history of these elements in their own right. Interestingly, we found that *Ty5*, a relatively rare retrotransposon with no previously known functional versions in *S. cerevisiae*, has retained functionality in the divergent East Asian Clade IX Complex. Additionally, we found that *Ty2*, a TE suggested to be a recent introduction to *S. cerevisiae* via *Saccharomyces mikatae* (Liti et al. 2005; Carr et al. 2012), is also present in the East Asian Clade IX Complex. This indicates that this event occurred early in *S. cerevisiae* history, that the donor–recipient relationship is reversed, that it happened multiply, or that this element was lost in *S. paradoxus* and other closely related species. With respect to the latter hypothesis, our genomic survey indicates numerous losses of functional different *Ty* elements in various strains suggesting that *Ty* extinction within clades is probably not uncommon and that near complete loss of all traces of extinct elements can occur relatively rapidly (see, e.g., *Ty4* and *Ty5* in fig. 4).

In conclusion, we suggest that the divergence of the East Asian Clade IX Complex occurred prior to the genetically close-knit, global radiation of *S. cerevisiae* strains we see today, potentially before their domestication. This begs the question whether there are truly wild *S. cerevisiae* strains outside of Asia at all, especially if the colonization of the rest of the world happened contemporarily with humans. Overall, this study generates new, valuable genomic resources and expands our understanding of the genetic variation and evolutionary history of one of the most important organisms in human history, *S. cerevisiae*. Moreover, this set of high-quality genomes, encompassing both domesticated and wild populations from different ecological backgrounds, provides an important resource for future explorations into the dynamics that govern eukaryotic genome evolution.

## Materials and Methods

### Yeast Strain Origins

We selected eight *S. cerevisiae* strains for long-read sequencing and genome assembly (table 1). Seven of these strains originate from East Asia. Six strains were isolated in China (Wang et al. 2012; Duan et al. 2018) from a variety of ecological niches and one in Taiwan (Peter et al. 2018). The six Chinese strains cover many of the lineages (CHN I, II, IV, VI, and IX) previously shown to be highly divergent from other *S. cerevisiae* strains based on short-read sequencing. The final strain (Y55) is a common laboratory strain isolated in France with a known mosaic genomic background originating in West Africa. To place our analyses in context, we also included currently publicly available *Saccharomyces* sensu stricto long-read genome assemblies as well as assemblies from *Torulaspora delbrueckii* and *Kluyveromyces lactis* (supplementary table S5, Supplementary Material online).

**Table 1:** Descriptions of the *Saccharomyces cerevisiae* Strains Sequenced in This Study

Lineage	Strain	Species	Source	Geographic Location
CHN I	HN1	*S. cerevisiae*	Rotten wood, primeval forest	Diaoluo Mountain, Hainan, China
CHN II	SX2	*S. cerevisiae*	Bark of a Fagaceae tree, primeval forest	Qinling Mountain, Shaanxi, China
CHN IV	HLJ1	*S. cerevisiae*	Bark of *Quercus mongokica*, secondary forest	Jingbo lake, Heilongjiang, China
CHN VI	BJ4	*S. cerevisiae*	Intestine of a butterfly, park	Haidian, Beijing, China
CHN IX	JXXY16.1	*S. cerevisiae*	Bark, primeval forest	Xiangxiyuan, Hubei Province, China
CHN IX	XXYS1.4	*S. cerevisiae*	Bark, primeval forest	Xiangxiyuan, Hubei Province, China
Taiwanese	EM14S01-3B	*S. cerevisiae*	Soil	Taiwan
West African+	Y55	*S. cerevisiae*	Wine grapes	France

### DNA Preparation and Long-Read Sequencing

Before sequencing, strains were sporulated and tetrads were dissected to allow for autodiploidization, making strains homozygous across all loci. Strains were incubated at 30 °C in 5 ml YEPD (1% yeast extract, 2% peptone, 2% dextrose) in a shaking incubator for 24 h before we harvested cells by centrifugation. We extracted genomic DNA using NucleoSpin Microbial DNA extraction kit according to the manufacturer’s instructions (Macherey-Nagel). Genomic DNA for strain Y55 was extracted independently using the QIAGEN Blood & Culture DNA Midi Kit. Samples were sequenced on PacBio Sequel and Sequel II platforms at the NGI/Uppsala Genome Center (Science for Life Laboratory, Sweden) and the University of Minnesota Sequencing Center (USA). In addition to these PacBio data, we also used publicly available paired-end Illumina sequence data previously generated for each strain (supplementary table S5, Supplementary Material online).

### Genome Assembly and Annotation

Nuclear contigs were assembled with Flye v2.8.1 (supplementary fig. S1, Supplementary Material online, default settings, est. genome size = 12.4 Mb) (Kolmogorov et al. 2019). We used short-read sequences for each strain to error-correct the long reads using FMLRC v2 (Wang et al. 2018). Corrected long reads and the short reads were subsequently used to polish the Flye assemblies using Racon v1.4.13 (Vaser et al. 2017) and POLCA v3.4.2 (Zimin and Salzberg 2020), respectively. We further scaffolded the contigs based on the reference S288C genome (GCA_000146045.2) using RaGOO v1.1 (Alonge et al. 2019) and filled any gaps this generated using multiple iterations of LR Gapcloser v1 (Xu et al. 2019) and Gapcloser (Luo et al. 2012). To account for any errors introduced by using long reads to fill gaps, we further polished each assembly once more using Racon v1.4.13 and POLCA v3.4.2. Mitochondrial assemblies were largely assembled using Flye without the assumption of even coverage (--metagenomic) using all long reads as input. JXXY16.1 and Y55 mitochondrial genomes were assembled using Flye v2.8.1 with default settings. Mitochondrial contigs were extracted by mapping the Flye output to the reference mitochondrial genome using Nucmer (Delcher et al. 2002). These assemblies were polished and scaffolded following the same process as that of the nuclear assemblies. Completeness of the final genome assemblies was assessed using BUSCO v4.0.5 (Simão et al. 2015; Waterhouse et al. 2018).

We annotated nuclear genes, mitochondrial genes, centromeres, TEs, core X elements, and Y-prime elements using modified versions of the pipelines within the LRSDAY package (Yue and Liti 2018). In addition to our eight newly assembled genomes, we also used the same method to annotate the previously published long-read assemblies (supplementary table S5, Supplementary Material online). Nuclear genes orthologous to annotated genes in the *S. cerevisiae* S288C reference genome were identified using Proteinortho v6.0.24 (Lechner et al. 2011). Genes for which no orthologous protein was found in the reference were clustered based on orthology to each other.

To further characterize *Ty* elements, we determined potential element viability by translating coding regions of full elements based on reading frames identified for each element in S288C. Elements containing premature stop codons or extensive frameshifts were categorized as putatively being reproductively inviable (loss of function). Additionally, we created gene trees for whole elements of each *Ty* class using MAFFT v7.471 alignments (default settings) with PhyML v3.0 (substitution model = HKY85; bootstrap = 100; tree searching using SRT and NNI; conducted in Unipro UGENE v36.0). To determine the likelihood of closely related elements within a given strain resulting from transposition or segmental genome duplication, we mapped the 10,000-bp regions containing each element to related intrastrain elements. To assess the differences in genomic content, we performed *t*-tests in the R environment using the t.test function (*t* = *t*-value indicating the size of difference relative to sample variation, df = degrees of freedom, sample size = 1).

The number of duplicated genes associated with each strain after a given node was determined using the sum of node duplications provided by our analysis with OrthoFinder (see below). We identified the number of candidate genes lost in a given *S. cerevisiae* clade as those genes existing in the cumulative gene set (i.e., pangenome) of all other *S. cerevisiae* strains that overlapped with the “pangenome” of the rest of our analyzed species, not present in the focal clade. Missing “core” genes were those genes present in the consensus set of the other of *S. cerevisiae* strains not found in the focal clade. For each set of gene losses, we determined the presence of an ortholog of the missing gene using the output of OrthoFinder (see below).

### Phylogenomic Analysis

To place our eight assembled genomes within the context of other *Saccharomyces* strains, we employed both a consensus gene tree and AAF approaches to phylogenetic tree construction. For consensus species trees, we used OrthoFinder v2.4.0 (fig. 1) in addition to a standard gene tree approach. For the latter, we aligned all orthologous genes found in at least five strains (5,847 genes) using MUSCLE v3.8.31 (Edgar 2004) and performed maximum-likelihood single-tree inference for each locus using RAxML-NG v1.01 (Kozlov et al. 2019) with a discrete GAMMA model of rate heterogeneity. We used Astral-III v5.7.4 (Zhang et al. 2018) with these gene trees to generate a consensus species tree.

AAF v20171001 (Fan et al. 2015) was used with a k-mer size of 20 nucleotides and a threshold frequency of 7 for each k-mer to be included in the analysis. AAF was used to compare the long-read and short-read sequencing data for the 25 *Saccharomyces* strains (supplementary fig. S2 and table S5, Supplementary Material online). Short-read sequencing data for *K. lactis* and *T. delbrueckii* were included as outgroups.

To generate the mitochondrial phylogeny, we reoriented the start of each assembly based on the position of the tRNA gene, trnP(ugg), then aligned these assemblies to each other using Mugsy v.1.2.3 (Angiuoli and Salzberg 2011). This multiple sequence alignment was then used to create a maximum likelihood tree using IQ-TREE v2.0.5 (options: -m TPM2u+F+R3 -B 1000 -bnni -alrt 1000) (Nguyen et al. 2015; Hoang et al. 2018). The model was determined using the ModelFinder component of IQ-Tree (Kalyaanamoorthy et al. 2017).

To ensure that the deep divergence identified between the East Asian Clade IX Complex relative to the rest of *S. cerevisiae* is not an artifact of highly divergent genes that originated via introgression, we generated a separate phylogeny with these regions removed from the analysis. Open reading frames (ORFs) with potential introgressive origins in the East Asian Clade IX Complex were identified by BLAST aligning each ORF of each Clade IX strain with its closest matching ORF in each non-Clade IX strain. Candidate-introgressed ORFs were determined as those found in all three Clade IX strains, with a mean percent identity relative to all other complementary *S. cerevisiae* ORFs ≤95%, for which the aligned region covered at least 75% of the query ORF, and for which two-thirds of alignments of all the Clade IX ORF alignments had no more than 95% identity. In total, we found 24 ORFs that passed these filters (supplementary table S6, Supplementary Material online).

### SV Detection

To identify the SVs between strains within *S. cerevisiae*, we performed exhaustive pairwise comparisons between the 15 strains with long-read assemblies (210 comparisons). We focused on five types of SV: deletions, insertions, tandem duplications, inversions, and translocations. The SVs were detected using MUM&Co (O’Donnell and Fischer 2020), which utilizes MUMmer v4 (Marçais et al. 2018) to perform whole-genome alignments and detect SVs ≥50 bp.

### Spore Viability Assay

To assess the level of reproductive isolation between the divergent East Asian strains and modern *S. cerevisiae*, we crossed all strains with Y55 (α; ho; leu2Δ::HygMX) (and Y55 to itself) and assessed the spore viability of each cross. We sporulated each strain by incubating them in liquid sporulation medium (KAC; 2% potassium acetate) for 3 days at 23 °C. These cultures were then incubated with 10 *μ*l zymolyase (100 U/ml) at 37 °C for 30 min before being plated on YEPD (2.5% agar) in equal mixture with cultures of Y55 (α; ho; leu2Δ::HygMX) and grown for 48 h at 30 °C. This culture was streaked on YEPD + hygromycin and replica plated to minimal media. A single colony was selected from each cross and grown up in liquid YEPD overnight, spun down, put in KAC, and incubated at room temperature with shaking for 4 days to induce sporulation. The resulting tetrads were treated with zymolyase for 30 min at room temperature. Five hundred microliters of sterile water was added before spores were dissected out of the tetrads onto YPD plates, using a Singer MSM 400 micromanipulator. We dissected 20 tetrads yielding 80 spores per cross. Plates were incubated at 30 °C and colonies were counted after 72 h, indicating viable spores that were able to germinate. To assess the differences in spore viability, we performed an ANOVA in the Python environment using the scipy.stats.f_oneway function (*F* = *F*-statistic indicating the variance between groups/variance within groups) (Virtanen et al. 2020).

Respiratory competence was determined by plating strains of yeast on rich media containing nonfermentable glycerol as the sole carbon source (1% yeast extract, 2% peptone, and 2% glycerol).

## Supplementary Material

Supplementary data are available at *Genome Biology and Evolution* online.

## Supplementary Material

evab001_Supplementary_Data
