# Corrigendum to: Genomic Evidence of an Ancient East Asian Divergence Event in Wild *Saccharomyces cerevisiae*

**DOI:** 10.1093/gbe/evab218

**Published:** 2021-10-06

**Authors:** Devin P Bendixsen, Noah Gettle, Ciaran Gilchrist, Zebin Zhang, Rike Stelkens

*Genome Biology and Evolution*, Volume 13, Issue 2, evab001, https://doi.org/10.1093/gbe/evab001

In the originally published version of this manuscript, Figure 3 and Figure 4 in the manuscript were swapped although the captions for each of the figures were correct and in the correct location. This has now been corrected.

