# Supplementary material for: Genomic Evidence of an Ancient East Asian Divergence Event in Wild Saccharomyces cerevisiae
Source: Genome Biol Evol. 2021 Jan 11;13(2):evab001. doi: 10.1093/gbe/evab001 (PMC7874999; doi:10.1093/gbe/evab001)
Supplement: evab001_Supplementary_Data [file evab001_supplementary_data.pdf]

Supplementary Material

Genomic evidence of an ancient East Asian divergence event in wild *Saccharomyces cerevisiae*

Devin P Bendixsen*, Noah Gettle*, Ciaran Gilchrist, Zebin Zhang, and Rike Stelkens

*These authors contributed equally to the study.

Division of Population Genetics, Department of Zoology, Stockholm University, Svante Arrheniusväg 18 B, 106 91 Stockholm, Sweden

Author for Correspondence: Devin P Bendixsen, Department of Zoology Stockholm University; email: devin.bendixsen@zoologi.su.se

Table S1: Metrics for nuclear and mitochondrial assemblies

	Metric	Y55	BJ4	HLJ1	HN1	SX2	EM14S01-3B	JXXY16.1	XXYS1.4
Nuclear	Mean read depth	99.65	165.91	45.31	66.14	174.18	76.84	25.96	76.46
	Total sequence count	16	16	16	16	16	16	16	16
	Total sequence length (bp)	11791742	11888108	11702338	11820137	11918758	11803938	11692380	11654158
	Min sequence length (bp)	230382	225758	229169	236734	226383	209147	223390	199212
	Max sequence length (bp)	1479087	1489054	1458639	1481187	1479650	1470812	1471675	1483244
	Mean sequence length (bp)	736983.88	743006.75	731396.12	738758.56	744922.38	737746.12	730773.75	728384.88
	Median sequence length (bp)	741368.5	752361.5	741606.5	758822	748579.5	755211	741203.5	733903.5
	N50 (bp)	916013	918555	895159	913524	928563	897577	902407	894632
	L50	6	6	6	6	6	6	6	6
	N90 (bp)	454735	426239	433662	434566	433099	444590	425488	432873
	L90	13	13	13	13	13	13	13	13
	GC%	38%	38%	38%	38%	38%	38%	38%	38%
	N%	0%	0%	0%	0%	0%	0%	0%	0%
	Genome size (Mbp)	11.79	11.89	11.7	11.82	11.92	11.8	11.69	11.65
Mitochondria	Total sequence count	1	1	1	1	1	1	1	1
	Total sequence length (bp)	80847	86123	78472	88103	80393	86728	68736	85245
	GC%	17%	15%	15%	14%	15%	15%	15%	15%
	N%	0%	0%	0%	0%	0%	0%	0%	0%

Table S2: Results of BUSCO genome completion analysis

Metric	Y55	BJ4	HLJ1	HN1	SX2	EM14S01-3B	JXXY16.1	XXYS1.4
Complete BUSCOs	98.7	98.5	98.2	98.4	98.5	98.5	97.6	96.5
Complete and single-copy BUSCOs	96.8	96.7	96.4	96.7	96.7	96.7	95.8	94.9
Complete and duplicated BUSCOs	1.9	1.8	1.8	1.7	1.8	1.8	1.8	1.6
Fragmented BUSCOs	0.1	0.1	0.2	0.1	0.2	0.1	0.3	0.9
Missing BUSCOs	1.2	1.4	1.6	1.5	1.3	1.4	2.1	2.6
Total BUSCO groups searched	2137	2137	2137	2137	2137	2137	2137	2137

Table S3: Genes present both within and outside of *S. cerevisiae* that are not present in the East Asian Clade IX Complex. Genes present in all non-East Asian Clade IX Complex strains are marked as 'core'.

gene	ortho	core
1351-augustus_masked-BBM_6-7257_1	F	F
APD1	F	T
APM4	F	T
ARC35	F	F
ARG1	F	F
ARG8	F	F
ARL1	F	T
ARO3	F	F
BIO5	F	T
BMH1	T	F
CHS2	F	F
CST6	F	F
CTF4	F	T
DGK1	F	T
DUS1	F	F
ENA2	T	F
ERG29	T	F
FCY21	T	F
FEN2	F	F
FMP41	F	T
FRS2	F	T
GG799_G0004940	F	F
GPI14	F	F
GPT2	F	T
GRX6	T	F
GSH2	F	F
HGH1	F	T
ICL1	F	T
INP52	T	F
LYP1	F	F
MAL12	T	F
MRPL51	T	F
NMD5	F	T
NOP10	F	F
PDS5	F	T
PER33	F	T
PHB2	F	T
PKH3	F	F
PLB1	T	F
PRP42	F	F
PRP5	F	T
QCR9	F	F
RPL7B	T	F
RPS12	T	T
RSR1	F	F
SAL1	T	F
SGA1	F	T
SKT5	F	F
SOR1	T	F
TAD3	F	F
TRM10	F	F
UBC12	F	F
VPS1	F	T
VPS75	F	T
YBL036C	F	T
YGL041W-A	F	F
YHR140W	F	F
YIL102C-A	T	T
YJU3	F	F
YPL229W	T	T
1473-maker-CET_4-8407_1	F	F
29-EC1118_1O4_6634g_1	F	F
528-maker-861-BGT_2_1	F	F
633-snap_masked-4265-BCN_8_1	F	F

gene	ortho	core
CR85_G0021510	F	F
CR85_G0026230	F	F
FAA3	F	F
PAU10	T	T
PAU15	T	F
PAU18	T	F
PCA1	T	T
TIR3	T	F
YCR025C	F	T
YCR041W	F	F
YJL222W-A	T	F
YKL036C	F	F
YKR073C	F	T
YMR084W	F	F
YOR029W	F	F
YRF1-1	T	F
87-augustus_masked-125-YCE_1	F	F
PAU1	T	F
PAU2	T	F
PAU4	T	F
PAU8	T	T
YAL067W-A	T	F
YBR013C	T	F
133-augustus_masked-1698-AIE_1_1	F	F
1515-maker-CPR_4-17889_1	F	F
1596-snap_masked-CPM_4-14907_1	F	F
209-augustus_masked-310-ALB_3_1	F	F
343-augustus_masked-772-BSG_4_1	F	F
489-maker-348-ALB_3_1	F	F
598-snap_masked-277-BFM_3_1	F	F
873-augustus_masked-YCC-11519_1	F	F
BJ4_G0027260	F	F
CBS432_G0005490	F	F
CBS432_G0014600	F	F
DBVPG6044_G0009690	F	F
DBVPG6044_G0034000	F	F
DBVPG6044_G0043860	F	F
HLJ1_G0014640	F	F
HN1_G0004060	F	F
NCYC3947_G0004910	F	F
NCYC3947_G0009890	F	F
NCYC3947_G0035770	F	F
RRT13	T	F
VTH1	T	F
YCR102W-A	T	F
YOR012W	T	F
ECM34	T	F
HLJ1_G0017100	F	F
YMR158C-A	T	F
147-augustus_masked-1985-AID_2_1	F	F
1628-snap_masked-CQS_4-6404_1	F	F
1730-snap_masked-BGQ_1-19910_1	F	F
849-augustus_masked-CCQ_1-5470_1	F	F
AHT1	F	F
BJ4_G0019790	F	F
BJ4_G0023980	F	F
BRP1	F	F
cox1-I5-orf346	F	F
ERR3	T	F
FLO9	T	F
HN1_G0048180	F	F
YEL028W	T	F
YER181C	T	F
YIL025C	F	F

Table S3 continued

gene	ortho	core
7621-YPL274W_1	F	F
CBS432_G0044490	F	F
ENA5	T	F
FEX2	T	F
FLR1	T	T
HXT6	T	F
IRC7	F	T
OAZ1	T	F
orf124	F	F
PHO92	F	F
RPL4A	T	F
TPK1	T	F
YFL066C	T	F
YGR079W	T	F
1083-augustus_masked-BPL_2-7584_1	F	F
1167-augustus_masked-CQS_4-6124_1	F	F
1644-snap_masked-BQN_4-5641_1	F	F
2289-YBR298C_NumOfGenes_2_1	F	F
30-EC1118_1O4_6645g_1	F	F
346-augustus_masked-779-BSG_4_1	F	F
7620-YPL273W_NumOfGenes_2_1	F	F
ARE1	F	F
atp8_1	F	F
BDS1	F	F
BJ4_G0038450	F	F
CBS12357_G0005860	F	F
CBS12357_G0012960	F	F
CBS12357_G0018350	F	F
CBS12357_G0018650	F	F
CBS12357_G0030710	F	F
CBS12357_G0052310	F	F
CBS12357_G0053210	F	F
CTF19	F	F
EMI1	T	F
GLO2	F	F
HAP2	F	T
LCB4	F	T
MATALPHA2	T	F
PAU20	T	F
QDR1	T	F
RTA1	T	T
SHE3	F	T
SOV1	F	F
SPC105	T	F
YCL021W-A	F	F
YET2	F	T
YIR020W-A	F	F
YNR066C	T	F
YSF3	F	F
YLL056C	T	T
1035-augustus_masked-CNP_1-20000_1	F	F
1127-augustus_masked-APS_4-8484_1	F	F
1205-augustus_masked-CGH_5-16625_1	F	F
156-augustus_masked-2151-BRV_4_1	F	F
1721-snap_masked-BDD_8-15410_1	F	F
1766-snap_masked-AEF_8-5136_1	F	F
4478-YHR216W_NumOfGenes_6_1	F	F
615-snap_masked-3161-AGR_2_1	F	F
AAD15	T	F
BJ4_G0051670	F	F
CR85_G0004590	F	F
CR85_G0004730	F	F
CR85_G0006320	F	F
CR85_G0015180	F	F

gene	ortho	core
YIL163C	T	T
YKL097C	F	F
771-augustus_masked-AKG_3-6639_1	F	F
CBS432_G0003020	F	F
CBS432_G0005580	F	F
CBS432_G0006310	F	F
CBS432_G0013440	F	F
CBS432_G0014970	F	F
CBS432_G0017300	F	F
CBS432_G0023340	F	F
CBS432_G0024030	F	F
CBS432_G0031000	F	F
CBS432_G0032260	F	F
CBS432_G0037500	F	F
CBS432_G0037910	F	F
CBS432_G0043550	F	F
ERR2	F	F
YAL069W	T	F
YBL113C	T	F
YDR157W	T	F
YER076W-A	F	F
YPR204W	T	F
YRF1-8	T	F
YLR462W	T	F
YRF1-5	T	F
1126-augustus_masked-APS_4-8484_1	F	F
144-augustus_masked-182-ABQ_6_1	F	F
1452-maker-YCR-6505_1	F	F
1497-maker-CDN_4-6216_1	F	F
1499-maker-BVC_6-19502_1	F	F
514-maker-5995-BBR_3_1	F	F
623-snap_masked-3817-CBB_4_1	F	F
BJ4_G0002660	F	F
DBVPG6044_G0006720	F	F
S288C_G0026350	F	F
S288C_G0040040	F	F
YBL044W	T	F
YGR051C	F	F
YHL045W	T	F
1980-YBL108W_1	F	F
YKR104W	T	F
1283-augustus_masked-BDM_5-23139_1	F	F
1677-snap_masked-AFA_4-7928_1	F	F
6395-YMR324C_1	F	F
BJ4_G0030300	F	F
BJ4_G0032400	F	F
DBVPG6044_G0029590	F	F
DBVPG6044_G0033190	F	F
DBVPG6765_G0053000	F	F
HLJ1_G0054650	F	F
PAU16	T	F
UFRJ50816_G0047770	F	F
YGL258W-A	F	F
YGL262W	T	F
YLR334C	T	F
YPL277C	T	F
YPL278C	T	F
59-augustus_masked-1113-BMC_1_1	F	F
AAD10	T	F
orf236	F	F
S288C_G0053880	F	F
UFRJ50816_G0023630	F	F
UFRJ50816_G0025530	F	F
UFRJ50816_G0025540	F	F
YIL177C	T	F

Table S4: Genes present in all *S. cerevisiae* strains outside of the East Asian Clade IX Complex with gene positions as found in strain SX1.

gene	orthologue present	found outside scer	chr	start	end
PAU8	T	T	chrI	11087	11449
YBL036C	F	T	chrII	155814	156587
FLR1	T	T	chrII	251561	253207
SHE3	F	T	chrII	488274	489551
APD1	F	T	chrII	537726	538676
ARL1	F	T	chrII	560537	561088
PRP5	F	T	chrII	687818	690367
PCA1	T	T	chrII	788514	792164
YCR022C	F	F	chrIII	156176	156520
YCR025C	F	T	chrIII	161697	162608
YDR194W-A	F	F	chrIV	828912	829064
PAU10	T	T	chrIV	1477897	1478259
YIL163C	T	T	chrIX	30882	31235
YIL102C-A	T	T	chrIX	165567	165794
SGA1	F	T	chrIX	170175	171824
ICL1	F	T	chrV	293273	294946
YER135C	F	F	chrV	448612	448914
FRS2	F	T	chrVI	110223	111734
IRC7	F	T	chrVI	273785	274987
HAP2	F	T	chrVII	60839	61639
HGH1	F	T	chrVII	860624	861808
RTA1	T	T	chrVII	909723	910676
PHB2	F	T	chrVII	947453	948385
YHR050W-A	F	F	chrVIII	204161	204331
NMD5	F	T	chrX	641089	644235
VPS1	F	T	chrXI	439434	441548
GPT2	F	T	chrXI	565651	567882
YKR073C	F	T	chrXI	575919	576161
YLL056C	T	T	chrXII	47435	48331
PER33	F	T	chrXII	278631	279452
YMR031W-A	F	F	chrXIII	322526	322792
YET2	F	T	chrXIII	338138	338620
PDS5	F	T	chrXIII	392404	396237
VPS75	F	T	chrXIV	188094	188983
FMP41	F	T	chrXIV	320639	321418
BIO5	F	T	chrXIV	724951	726546
APM4	F	T	chrXV	220700	222175
YOL019W-A	T	F	chrXV	297602	297772
LCB4	F	T	chrXV	658425	660299
DGK1	F	T	chrXV	898395	899267
YOR343C	T	F	chrXV	967600	967827
RPS12	T	T	chrXV	1020909	1021340
YOR376W	F	F	chrXV	1035900	1036268
YPL229W	T	T	chrXVI	111893	112558
CTF4	F	T	chrXVI	786727	789510

Table S5: Information about long and short-read sequencing datasets used in this study.

Genome	Species	Long-read Sequencing			Short-read Sequencing		
		Source	Instrument	ENA Accession	Source	Instrument	ENA Accession
HN1	S. cerevisiae	**this study**	PacBio Sequel	PRJEB38713	Duan et al. 2018	Illumina HiSeq 2000	PRJNA396809
SX2	S. cerevisiae	**this study**	PacBio Sequel	PRJEB38713	Duan et al. 2018	Illumina HiSeq 2000	PRJNA396809
HLJ1	S. cerevisiae	**this study**	PacBio Sequel	PRJEB38713	Duan et al. 2018	Illumina HiSeq 2000	PRJNA396809
BJ4	S. cerevisiae	**this study**	PacBio Sequel	PRJEB38713	Duan et al. 2018	Illumina HiSeq 2000	PRJNA396809
JXXY16.1	S. cerevisiae	**this study**	PacBio Sequel	PRJEB38713	Duan et al. 2018	Illumina HiSeq 2000	PRJNA396809
XXYS1.4	S. cerevisiae	**this study**	PacBio Sequel	PRJEB38713	Duan et al. 2018	Illumina HiSeq 2000	PRJNA396809
EM14S01-3B	S. cerevisiae	**this study**	PacBio Sequel	PRJEB38713	Peter et al. 2018	Illumina HiSeq 2000	PRJEB13017
Y55	S. cerevisiae	**this study**	PacBioSequel	PRJEB38713	NA	Illumina HiSeq 4000	PRJNA552112
S288C	S. cerevisiae	Yue et al. 2017	PacBio RS	PRJEB7245	Yue et al. 2017	Illumina HiSeq 2500	PRJNA340312
DBVPG6044	S. cerevisiae	Yue et al. 2017	PacBio RS	PRJEB7245	Yue et al. 2017	Illumina HiSeq 2500	PRJNA340312
DBVPG6765	S. cerevisiae	Yue et al. 2017	PacBio RS	PRJEB7245	Yue et al. 2017	Illumina HiSeq 2500	PRJNA340312
SK1	S. cerevisiae	Yue et al. 2017	PacBio RS	PRJEB7245	Yue et al. 2017	Illumina HiSeq 2500	PRJNA340312
Y12	S. cerevisiae	Yue et al. 2017	PacBio RS	PRJEB7245	Yue et al. 2017	Illumina HiSeq 2500	PRJNA340312
YPS128	S. cerevisiae	Yue et al. 2017	PacBio RS	PRJEB7245	Yue et al. 2017	Illumina HiSeq 2500	PRJNA340312
UWOPS03-461.4	S. cerevisiae	Yue et al. 2017	PacBio RS	PRJEB7245	Yue et al. 2017	Illumina HiSeq 2500	PRJNA340312
CBS432	S. paradoxus	Yue et al. 2017	PacBio RS	PRJEB7245	Yue et al. 2017	Illumina HiSeq 2500	PRJNA340312
N44	S. paradoxus	Yue et al. 2017	PacBio RS	PRJEB7245	Yue et al. 2017	Illumina HiSeq 2500	PRJNA340312
YPS138	S. paradoxus	Yue et al. 2017	PacBio RS	PRJEB7245	Yue et al. 2017	Illumina HiSeq 2500	PRJNA340312
UFRJ50816	S. paradoxus	Yue et al. 2017	PacBio RS	PRJEB7245	Yue et al. 2017	Illumina HiSeq 2500	PRJNA340312
UWOPS91-917.1	S. paradoxus	Yue et al. 2017	PacBio RS	PRJEB7245	Yue et al. 2017	Illumina HiSeq 2500	PRJNA340312
NCYC3947	S. jurei	Naseeb et al. 2018	PacBio RS II	PRJEB24816	Naseeb et al. 2018	Illumina HiSeq 2500	PRJEB24816
CR85	S. kudriavzevii	Macías et al. 2019	Roche 454	PRJEB31099	Boonekamp et al. 2018	Illumina HiSeq 2500	PRJNA480800
CBS12357	S. eubayanus	NA	Nanopore MinION	PRJNA450912	Hebly et al. 2015	Illumina HiSeq 2500	PRJNA450912
COFT1	T. delbrueckii	NA	Nanopore MinION	PRJNA435462	Gomez-Angulo et al. 2015	Genome Analyzer IIx	PRJNA278774
GG799	K. lactis	NA	PacBio	PRJNA386037	NA	NA	NA

Table S6: Candidate introgressed ORFs in East Asian Clade IX Complex strains

ORF	Strain	Chromosome	Start	End	Best match strain	Best match species	Percent identity (relative to best match)
YIL086C	**EM14S01-3B**	chrIX	203713	204030	BJ4	S. cerevisiae	94.565
TIR1	**EM14S01-3B**	chrV	179192	179938	YPS128	S. cerevisiae	95.448
RRT5	**EM14S01-3B**	chrVI	233435	234265	HN1	S. cerevisiae	95.793
VEL1	**EM14S01-3B**	chrVII	9129	9749	HLJ1	S. cerevisiae	93.579
DBVPG6044_G0027530	**EM14S01-3B**	chrVIII	454606	454851	DBVPG6765	S. cerevisiae	94.667
YAR053W	**EM14S01-3B**	chrVIII	529062	529358	HN1	S. cerevisiae	95.849
YKL183C-A	**EM14S01-3B**	chrXI	98497	98709	SX2	S. cerevisiae	96.714
OAF3	**EM14S01-3B**	chrXI	564680	567277	CBS432	S. paradoxus	92.879
PAM17	**EM14S01-3B**	chrXI	567427	568020	CBS432	S. paradoxus	93.771
CCP1	**EM14S01-3B**	chrXI	568239	569321	N44	S. paradoxus	93.813
MET1	**EM14S01-3B**	chrXI	574038	575813	N44	S. paradoxus	94.2
YKR070W	**EM14S01-3B**	chrXI	575999	577057	YPS138	S. paradoxus	93.484
DRE2	**EM14S01-3B**	chrXI	577364	578410	N44	S. paradoxus	92.932
SIS2	**EM14S01-3B**	chrXI	578852	580477	CBS432	S. paradoxus	89.006
FKS1	**EM14S01-3B**	chrXII	754638	760268	HN1	S. cerevisiae	98.863
MSA2	**EM14S01-3B**	chrXII	1007990	1009090	SX2	S. cerevisiae	83.008
YKR078W	**EM14S01-3B**	chrXII	1009297	1011045	N44	S. paradoxus	89.692
RSN1	**EM14S01-3B**	chrXIII	771948	774803	HLJ1	S. cerevisiae	99.72
RSN1	**EM14S01-3B**	chrXIII	771948	774803	YPS128	S. cerevisiae	99.72
YNL277W-A	**EM14S01-3B**	chrXIV	105287	105466	DBVPG6765	S. cerevisiae	93.989
AGA1	**EM14S01-3B**	chrXIV	683249	685489	SK1	S. cerevisiae	95.63
TIR4	**EM14S01-3B**	chrXV	325197	326510	YPS128	S. cerevisiae	98.655
YOR376W-A	**EM14S01-3B**	chrXV	1014840	1014995	SX2	S. cerevisiae	94.231
FIT3	**EM14S01-3B**	chrXV	1029603	1030226	SK1	S. cerevisiae	96.491
YIL086C	**JXXY16.1**	chrIX	192734	193051	BJ4	S. cerevisiae	94.737
TIR1	**JXXY16.1**	chrV	175973	176719	YPS128	S. cerevisiae	95.315
RRT5	**JXXY16.1**	chrVI	229468	230298	HN1	S. cerevisiae	95.553
VEL1	**JXXY16.1**	chrVII	5075	5695	HLJ1	S. cerevisiae	93.579
DBVPG6044_G0027530	**JXXY16.1**	chrVIII	443172	443417	DBVPG6765	S. cerevisiae	95.333
YAR053W	**JXXY16.1**	chrVIII	518612	518908	HN1	S. cerevisiae	96.226
YKL183C-A	**JXXY16.1**	chrXI	98975	99187	SX2	S. cerevisiae	96.714
OAF3	**JXXY16.1**	chrXI	562341	564938	CBS432	S. paradoxus	92.841
PAM17	**JXXY16.1**	chrXI	565088	565681	CBS432	S. paradoxus	93.771
CCP1	**JXXY16.1**	chrXI	565900	566982	N44	S. paradoxus	93.721
MET1	**JXXY16.1**	chrXI	571710	573485	N44	S. paradoxus	94.257
YKR070W	**JXXY16.1**	chrXI	573671	574729	YPS138	S. paradoxus	93.484
DRE2	**JXXY16.1**	chrXI	575036	576082	N44	S. paradoxus	92.741
SIS2	**JXXY16.1**	chrXI	576524	578149	CBS432	S. paradoxus	89.006
FKS1	**JXXY16.1**	chrXII	752029	757659	HN1	S. cerevisiae	98.917
MSA2	**JXXY16.1**	chrXII	996435	997535	SX2	S. cerevisiae	83.008
YKR078W	**JXXY16.1**	chrXII	997742	999490	N44	S. paradoxus	89.806
RSN1	**JXXY16.1**	chrXIII	767842	770697	HLJ1	S. cerevisiae	99.755
RSN1	**JXXY16.1**	chrXIII	767842	770697	YPS128	S. cerevisiae	99.755
YNL277W-A	**JXXY16.1**	chrXIV	103284	103463	DBVPG6765	S. cerevisiae	93.989
AGA1	**JXXY16.1**	chrXIV	683593	685686	Y12	S. cerevisiae	95.672
TIR4	**JXXY16.1**	chrXV	340503	341816	YPS128	S. cerevisiae	98.655
YOR376W-A	**JXXY16.1**	chrXV	1019031	1019186	SX2	S. cerevisiae	94.872
FIT3	**JXXY16.1**	chrXV	1034210	1034830	SK1	S. cerevisiae	96.491
YIL086C	**XXYS1.4**	chrIX	199474	199791	BJ4	S. cerevisiae	94.565
TIR1	**XXYS1.4**	chrV	181244	181990	YPS128	S. cerevisiae	95.448
RRT5	**XXYS1.4**	chrVI	222357	223187	HN1	S. cerevisiae	95.553
VEL1	**XXYS1.4**	chrVII	10008	10628	HLJ1	S. cerevisiae	93.579
DBVPG6044_G0027530	**XXYS1.4**	chrVIII	446504	446749	DBVPG6765	S. cerevisiae	95.333
YAR053W	**XXYS1.4**	chrVIII	522431	522727	HN1	S. cerevisiae	96.226
YKL183C-A	**XXYS1.4**	chrXI	99813	100025	SX2	S. cerevisiae	96.714
OAF3	**XXYS1.4**	chrXI	561164	563761	CBS432	S. paradoxus	92.841
PAM17	**XXYS1.4**	chrXI	563911	564504	CBS432	S. paradoxus	93.771
CCP1	**XXYS1.4**	chrXI	564723	565805	N44	S. paradoxus	93.813
MET1	**XXYS1.4**	chrXI	570531	572306	N44	S. paradoxus	94.2
YKR070W	**XXYS1.4**	chrXI	572492	573550	YPS138	S. paradoxus	93.39
DRE2	**XXYS1.4**	chrXI	573857	574903	N44	S. paradoxus	92.932
SIS2	**XXYS1.4**	chrXI	575345	576826	GG799	K. lactis	94.737
FKS1	**XXYS1.4**	chrXII	761195	766825	HN1	S. cerevisiae	98.917
MSA2	**XXYS1.4**	chrXII	1005393	1006493	SX2	S. cerevisiae	83.008
YKR078W	**XXYS1.4**	chrXII	1006700	1008448	N44	S. paradoxus	89.692
RSN1	**XXYS1.4**	chrXIII	775890	778745	HLJ1	S. cerevisiae	99.72
RSN1	**XXYS1.4**	chrXIII	775890	778745	YPS128	S. cerevisiae	99.72
YNL277W-A	**XXYS1.4**	chrXIV	100600	100779	DBVPG6765	S. cerevisiae	93.989
AGA1	**XXYS1.4**	chrXIV	666457	668739	SX2	S. cerevisiae	95.316
TIR4	**XXYS1.4**	chrXV	314551	315864	YPS128	S. cerevisiae	98.804
YOR376W-A	**XXYS1.4**	chrXV	990842	990997	SX2	S. cerevisiae	94.872
FIT3	**XXYS1.4**	chrXV	1005357	1005830	SK1	S. cerevisiae	95.402

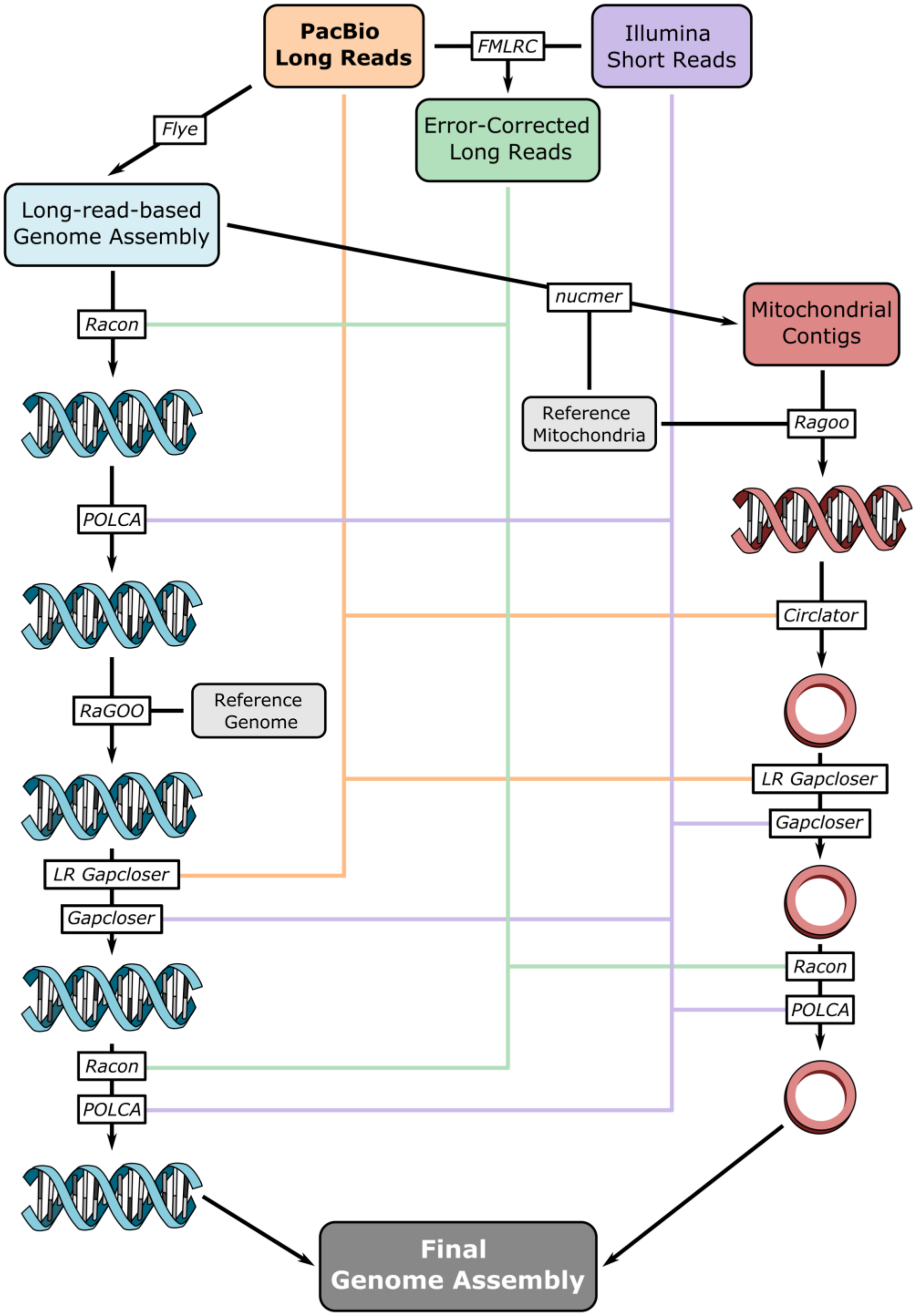

Figure S1: Nuclear and mitochondrial genome assembly pipeline. Workflow for assembly of nuclear (blue) and mitochondrial (red) genomes. PacBio long reads generated in this study are indicated in orange, while publicly available short-reads (**Table S3**) are indicated in purple. It is important to note that the Flye assembly processes were independent for nuclear and mitochondrial assembly, as indicated in the methods. The reference nuclear and mitochondrial genome used for assembly was S288C.

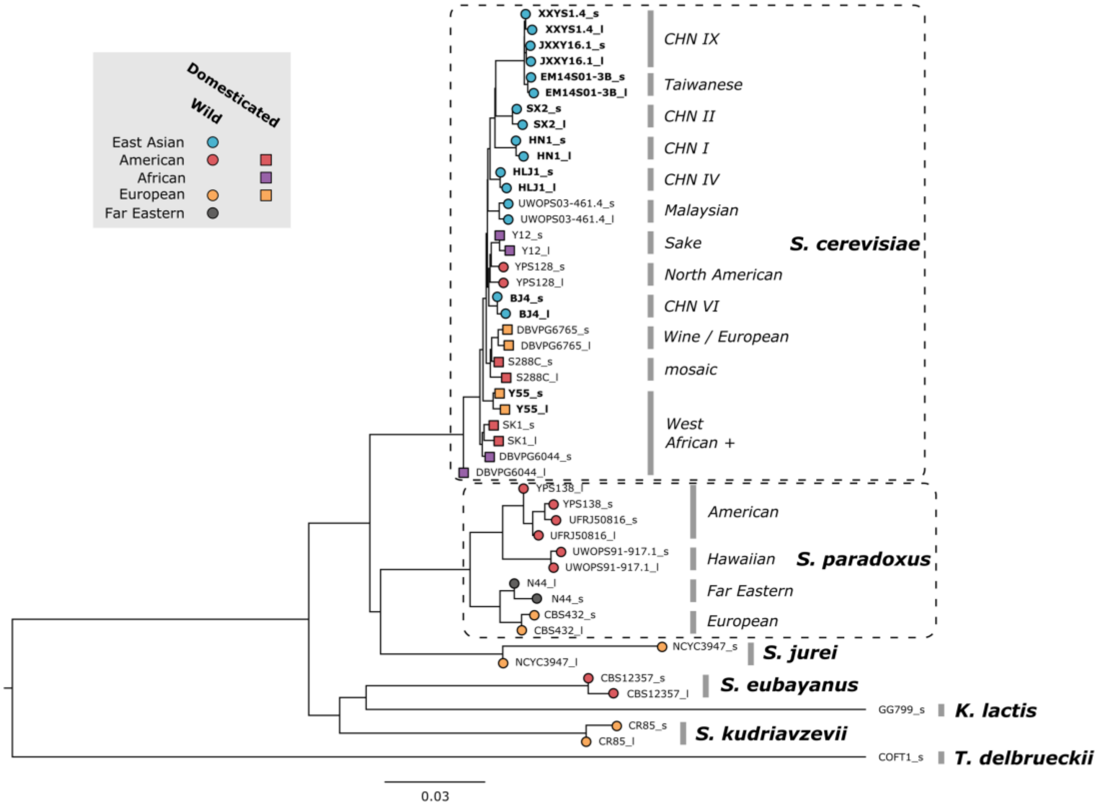

Figure S2: Alignment and Assembly-Free (AAF) phylogenetic tree of short and long-read sequencing data Phylogenetic network generated using an Alignment and Assembly-Free (AAF) approach. The analysis was performed with k-mer size of 20 and a threshold frequency of 7 for each k-mer to be included in the analysis. For species with more than a single long-read genome assembly (*S. cerevisiae and S. paradoxus*), species clades are indicated in italics. *Saccharomyces* strains are colored according to their location of origin and branch tip shape indicates whether it is a domesticated (square) or wild (circle) strain. New long-read sequencing data presented in this study are indicated in bold. Short-read and long-read sequencing data are labeled '_s' and '_l' respectively, following the strain name.

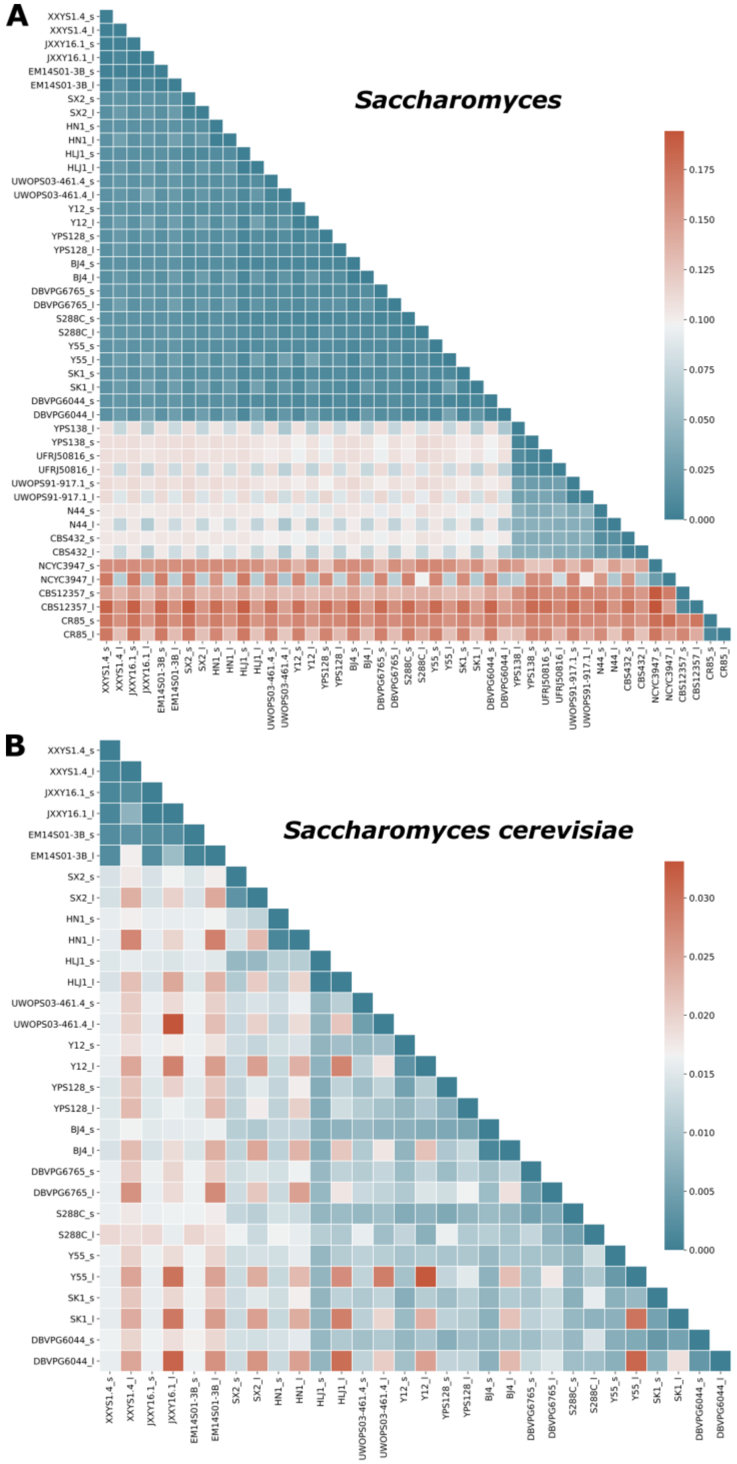

Figure S3: Assembly and Alignment Free (AAF) Distance matrix of short-read vs long-read (A) Pairwise distance matrix generated from AAF of short read and long-read sequencing data. Short-read and long-read sequencing data are labeled '_s' and '_l' respectively, following the strain name. The analysis was performed with k-mer size of 20 and a threshold frequency of 7 for each k-mer to be included in the analysis. **(B)** A closer look at only *S. cerevisiae* strains.

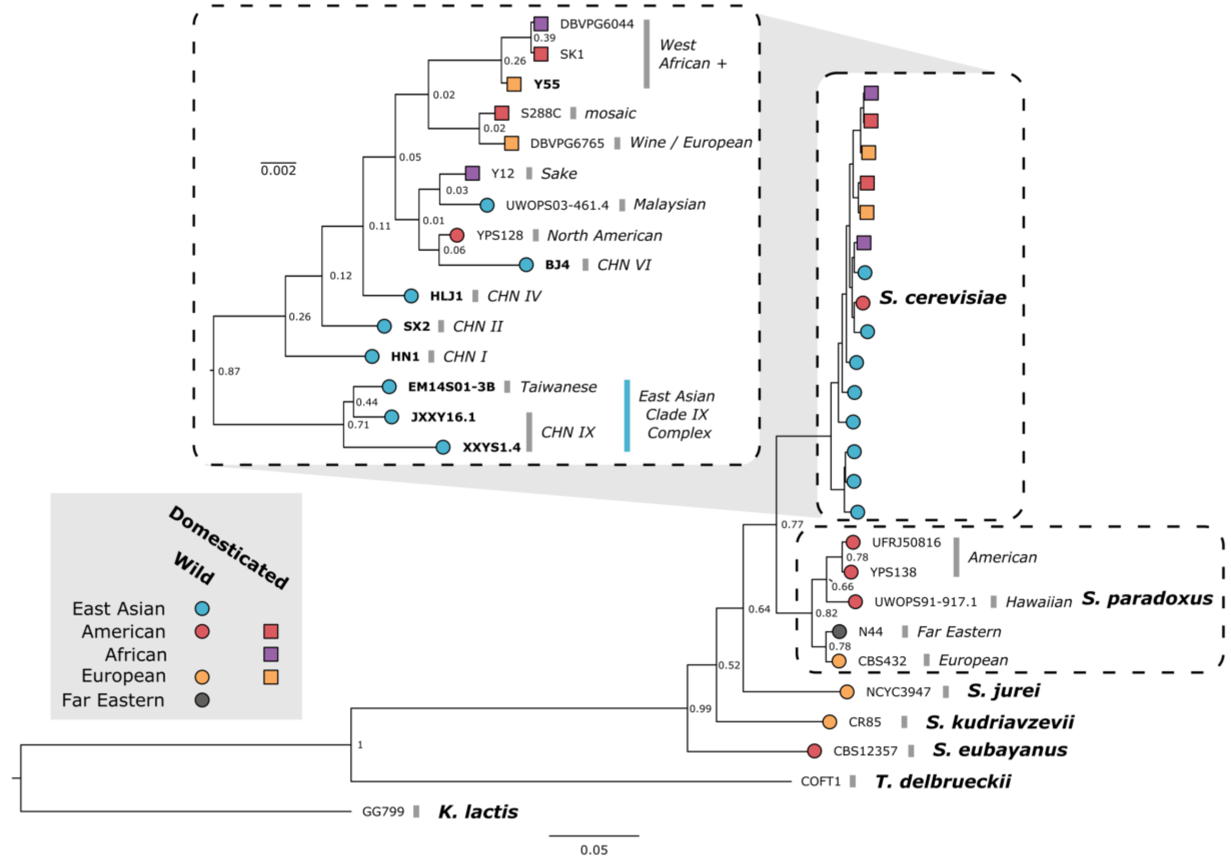

Figure S4: Consensus phylogenetic tree of yeast long-read genomes without introgressed genes. The tree was built identically to the tree in **Fig. 1** using orthogroup inference, however the genes that were identified as potentially introgressed in the East Asian Clade IX Complex (Table S6) were excluded. The support values are the proportion of times that the bipartition is seen in each of the individual species tree estimates. Branch lengths represent the average number of substitutions per site across the sampled gene families. For species with more than a single long-read genome assembly (*S. cerevisiae and S. paradoxus*), species clades are indicated in italics. Strains are colored according to their location of origin and branch tip shape indicates whether it is domesticated (square) or wild (circle). Inset depicts *S. cerevisiae* strains with independent scaling. New long-read genome assemblies presented in this study are indicated in bold.

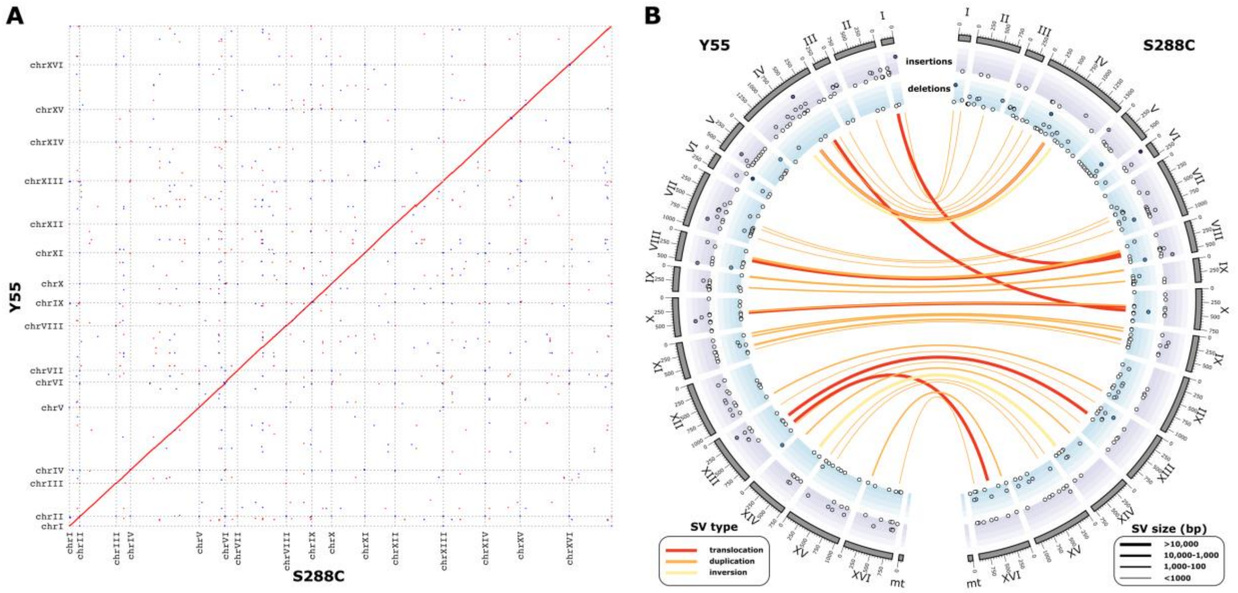

Figure S5: Y55 genome assembly (A) Genome comparison of the reference strain, S288C, and Y55. Sequence homology within the dot plots is indicated by red dots for forward matches and blue dots for reverse matches. **(B)** CIRCOS plot showing the detected structural variations between reference strain, S288C and Y55. Translocations (red), duplications (orange) and inversions (yellow) are depicted as links between the two genomes. The width of the link reflects the relative size of the variation (bp). Insertions (blue) and deletions (purple) are depicted in the outer tracks. Deletion and insertion size increase towards the outside. Chromosome size is depicted on the outside in 1kb units.

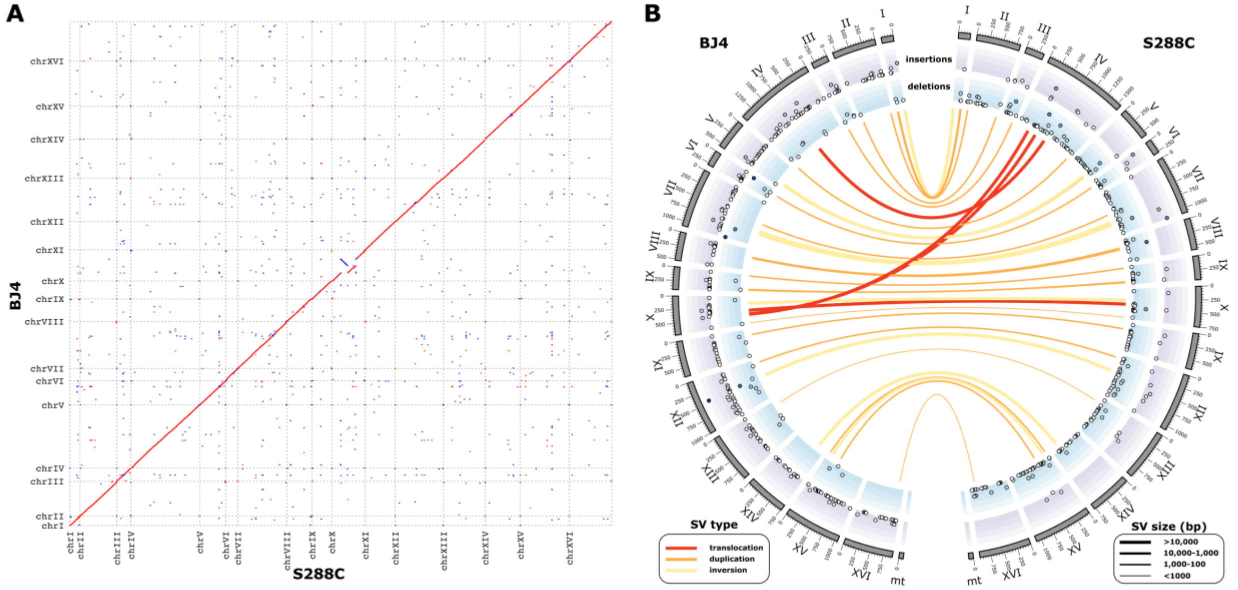

Figure S6: BJ4 genome assembly (A) Genome comparison of the reference strain, S288C, and BJ4. Sequence homology within the dot plots is indicated by red dots for forward matches and blue dots for reverse matches. **(B)** CIRCOS plot showing the detected structural variations between reference strain, S288C and BJ4. Translocations (red), duplications (orange) and inversions (yellow) are depicted as links between the two genomes. The width of the link reflects the relative size of the variation (bp). Insertions (blue) and deletions (purple) are depicted in the outer tracks. Deletion and insertion size increase towards the outside. Chromosome size is depicted on the outside in 1kb units.

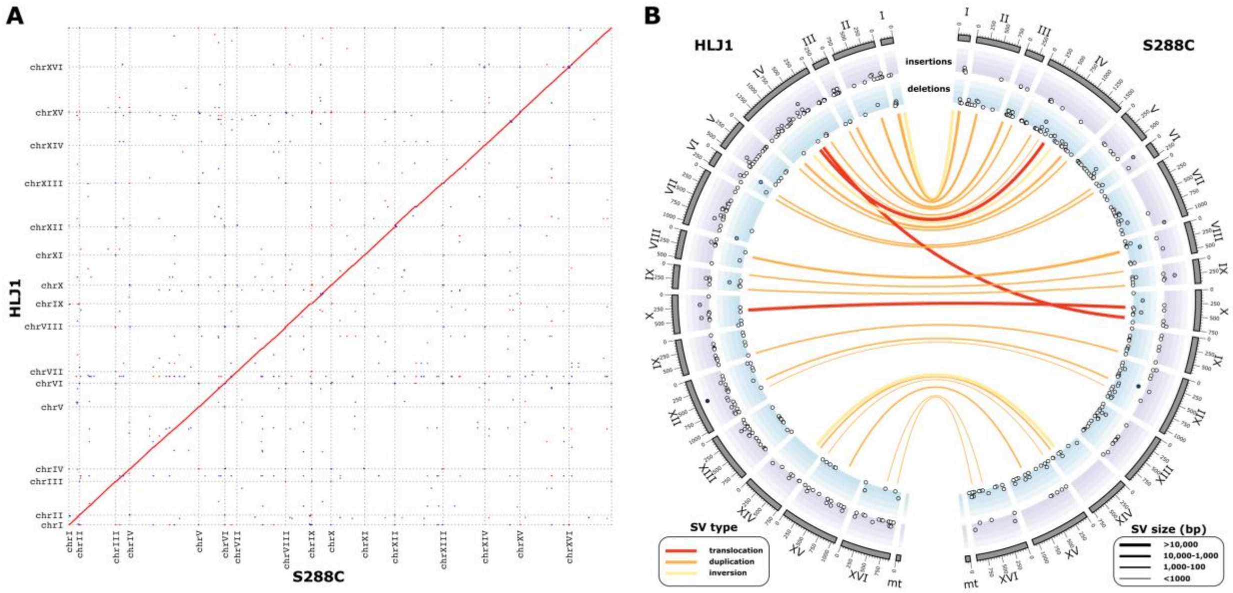

Figure S7: HLJ1 genome assembly (A) Genome comparison of the reference strain, S288C, and HLJ1. Sequence homology within the dot plots is indicated by red dots for forward matches and blue dots for reverse matches. **(B)** CIRCOS plot showing the detected structural variations between reference strain, S288C and HLJ1. Translocations (red), duplications (orange) and inversions (yellow) are depicted as links between the two genomes. The width of the link reflects the relative size of the variation (bp). Insertions (blue) and deletions (purple) are depicted in the outer tracks. Deletion and insertion size increase towards the outside. Chromosome size is depicted on the outside in 1kb units

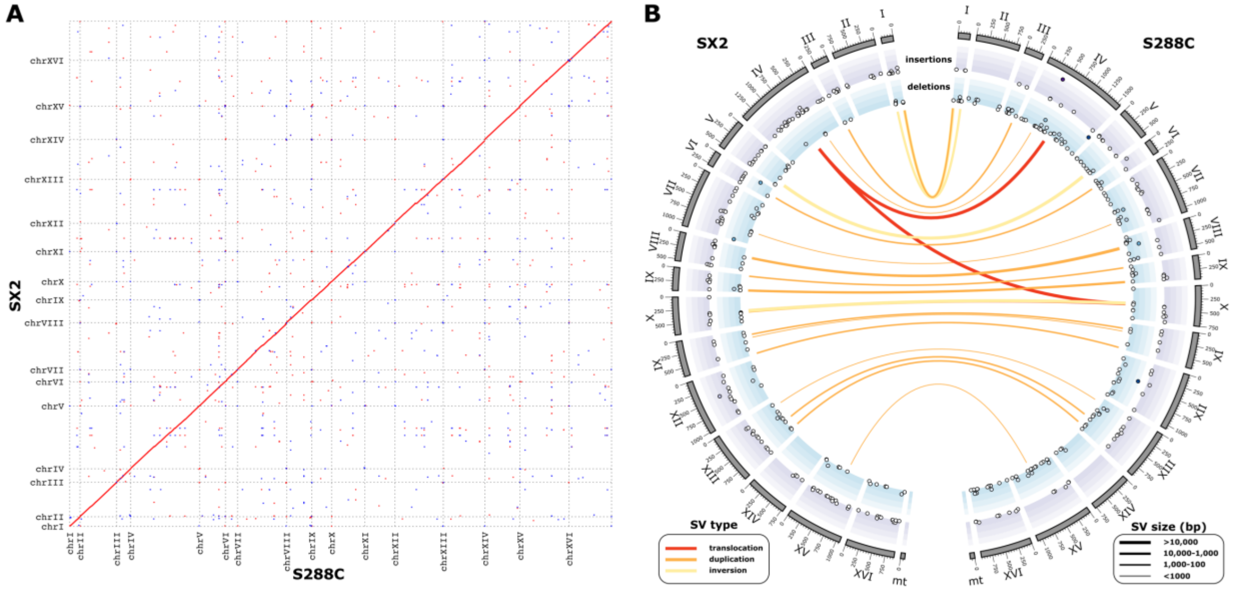

Figure S8: SX2 genome assembly (A) Genome comparison of the reference strain, S288C, and SX2. Sequence homology within the dot plots is indicated by red dots for forward matches and blue dots for reverse matches. **(B)** CIRCOS plot showing the detected structural variations between reference strain, S288C and SX2. Translocations (red), duplications (orange) and inversions (yellow) are depicted as links between the two genomes. The width of the link reflects the relative size of the variation (bp). Insertions (blue) and deletions (purple) are depicted in the outer tracks. Deletion and insertion size increase towards the outside. Chromosome size is depicted on the outside in 1kb units.

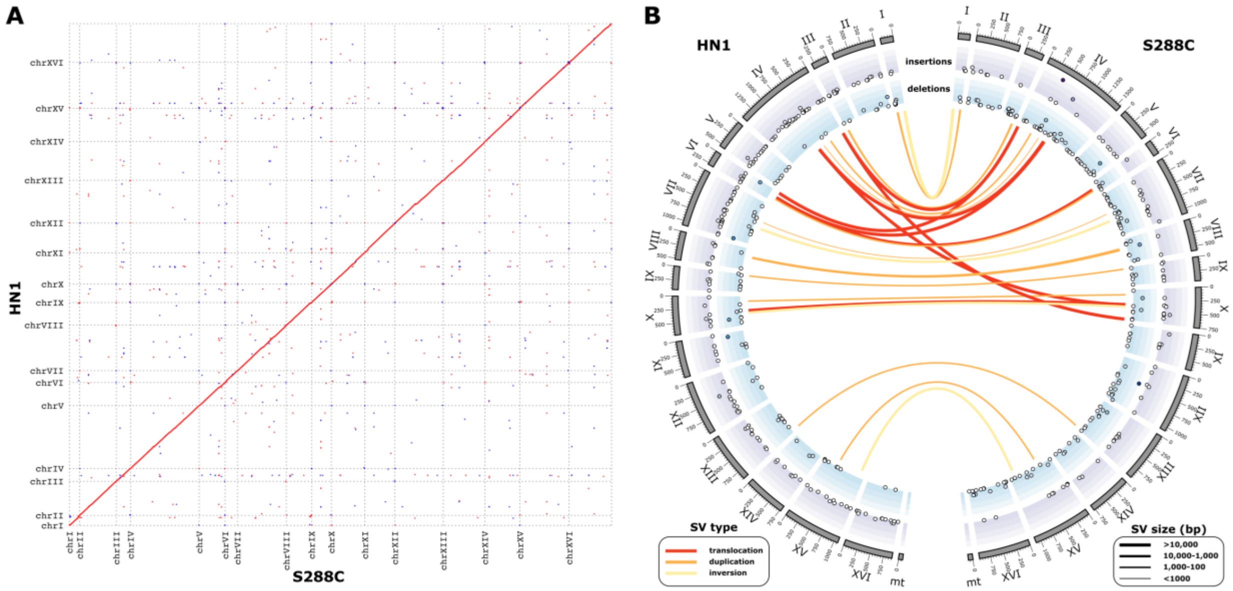

Figure S9: HN1 genome assembly (A) Genome comparison of the reference strain, S288C, and HN1. Sequence homology within the dot plots is indicated by red dots for forward matches and blue dots for reverse matches. **(B)** CIRCOS plot showing the detected structural variations between reference strain, S288C and HN1. Translocations (red), duplications (orange) and inversions (yellow) are depicted as links between the two genomes. The width of the link reflects the relative size of the variation (bp). Insertions (blue) and deletions (purple) are depicted in the outer tracks. Deletion and insertion size increase towards the outside. Chromosome size is depicted on the outside in 1kb units.

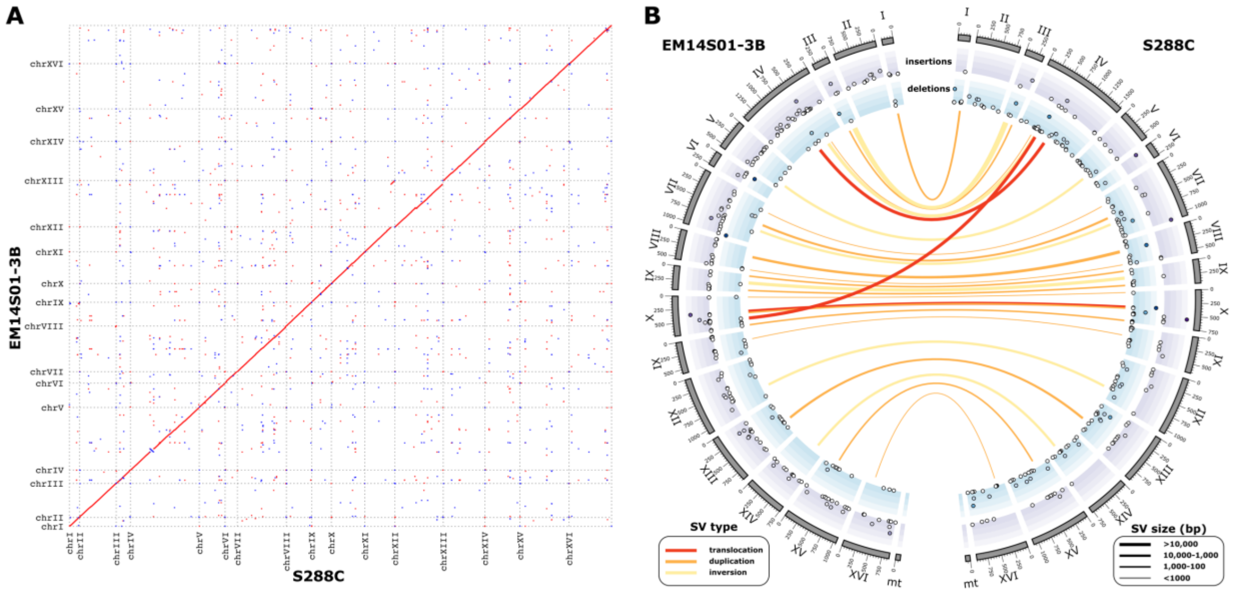

Figure S10: EM14S01-3B genome assembly (A) Genome comparison of the reference strain, S288C, and EM14S01-3B. Sequence homology within the dot plots is indicated by red dots for forward matches and blue dots for reverse matches. **(B)** CIRCOS plot showing the detected structural variations between reference strain, S288C and EM14S01-3B. Translocations (red), duplications (orange) and inversions (yellow) are depicted as links between the two genomes. The width of the link reflects the relative size of the variation (bp). Insertions (blue) and deletions (purple) are depicted in the outer tracks. Deletion and insertion size increase towards the outside. Chromosome size is depicted on the outside in 1kb units.

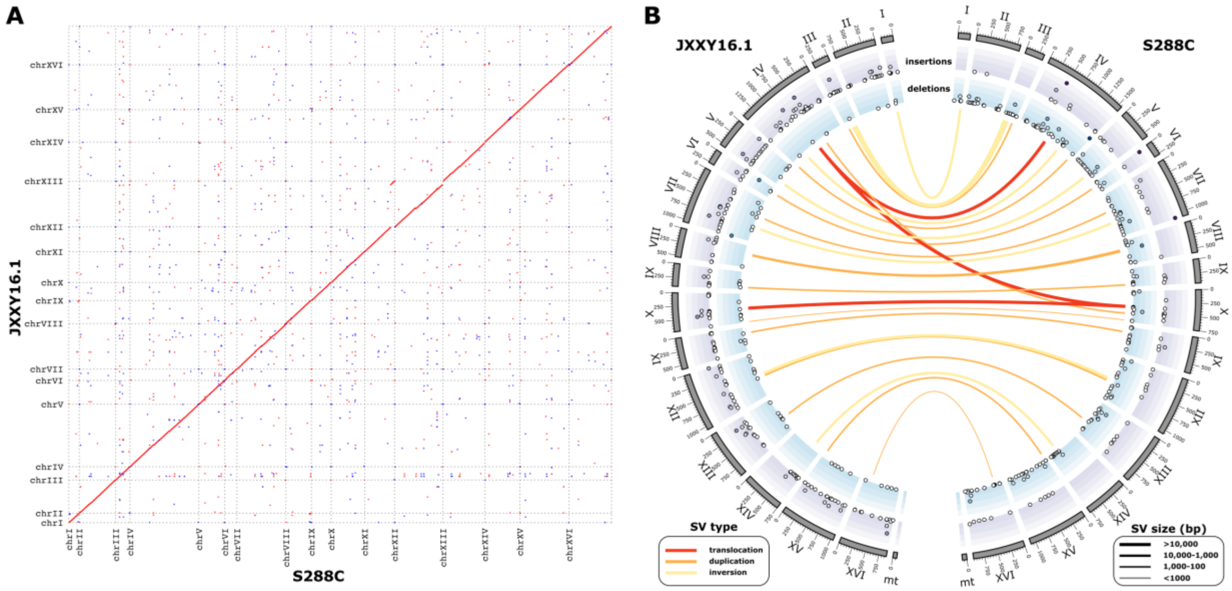

Figure S11: JXXY16.1 genome assembly (A) Genome comparison of the reference strain, S288C, and JXXY16.1. Sequence homology within the dot plots is indicated by red dots for forward matches and blue dots for reverse matches. **(B)** CIRCOS plot showing the detected structural variations between reference strain, S288C and JXXY16.1. Translocations (red), duplications (orange) and inversions (yellow) are depicted as links between the two genomes. The width of the link reflects the relative size of the variation (bp). Insertions (blue) and deletions (purple) are depicted in the outer tracks. Deletion and insertion size increase towards the outside. Chromosome size is depicted on the outside in 1kb units.

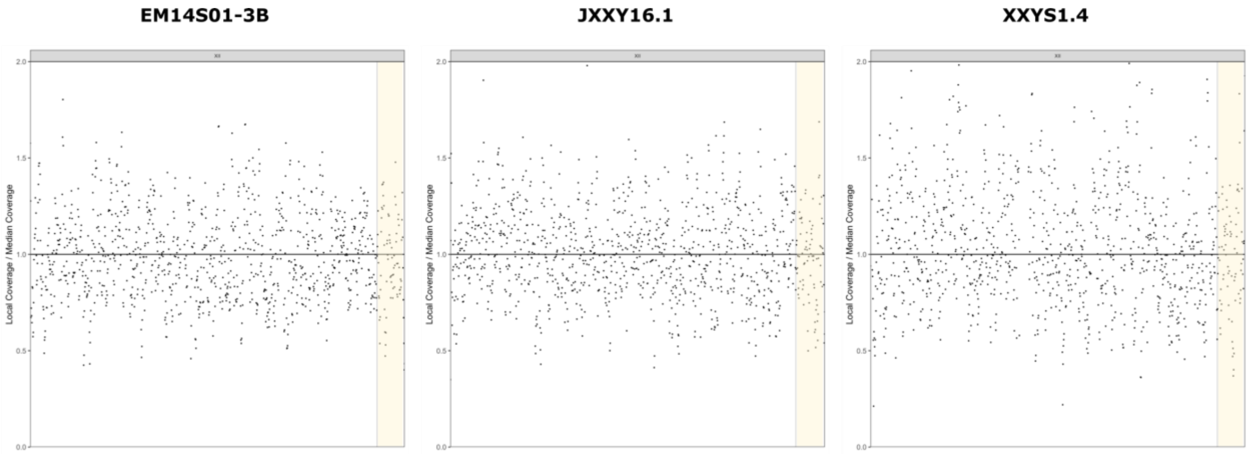

Figure S12: Long-read sequencing coverage of chromosome XII in East Asian Clade IX strains. X-axis indicates the length of the chromosome. Y-axis indicates the binned local coverage/ median coverage. Highlighted yellow region indicates the portion of the chromosome translocated from chromosome XI.

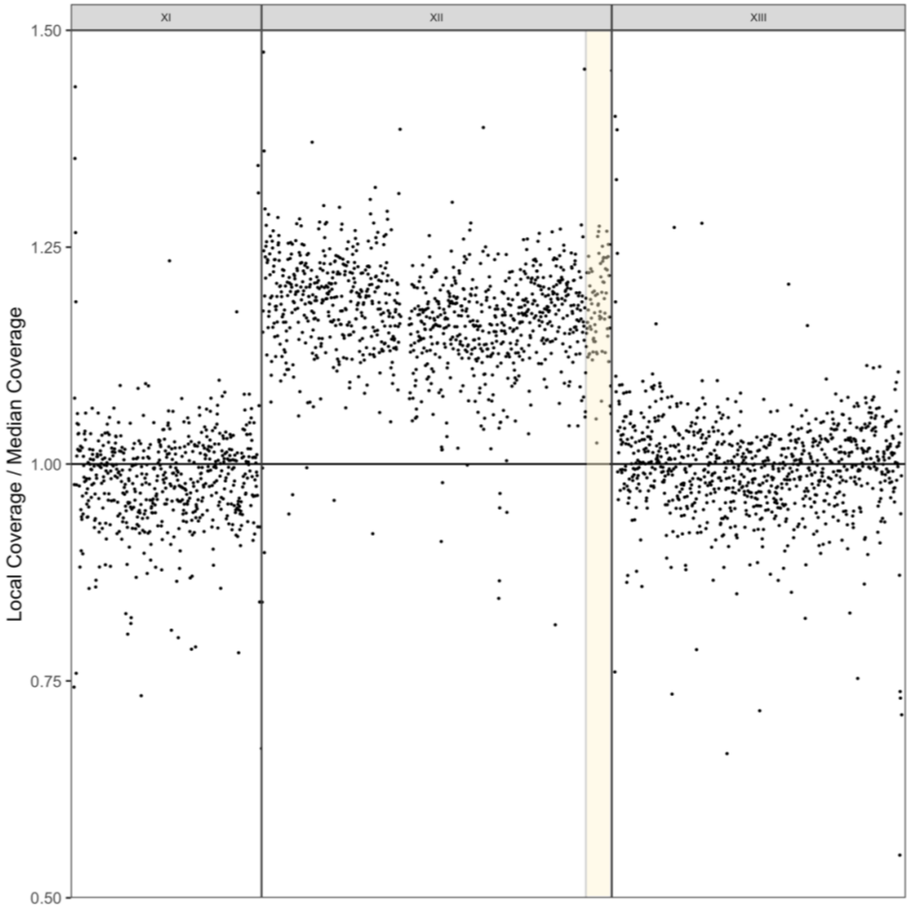

Figure S13: Short-read sequencing coverage of large translocation in XXYS1.4. X-axis indicates the length of the chromosome. Y-axis indicates the binned local coverage/ median coverage. Highlighted yellow region indicates the portion of the chromosome translocated from chromosome XI.

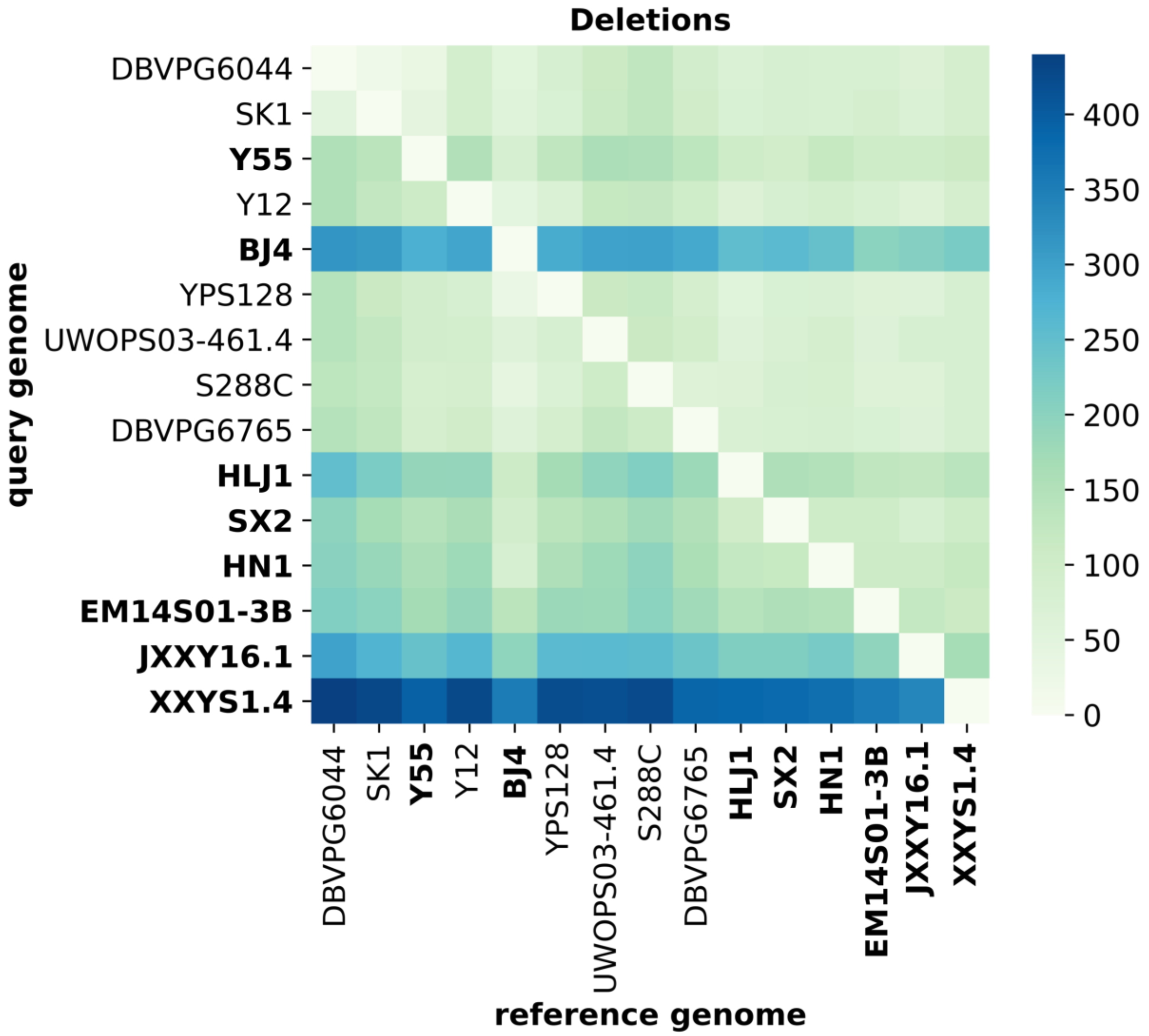

Figure S14: Deletions within *Saccharomyces cerevisiae*. Pairwise comparisons among all *Saccharomyces cerevisiae* genome assemblies depicting the total number of variations. Order of genome assemblies is consistent with the species tree (**Fig. 1**). New long-read genome assemblies presented in this study are indicated in bold.

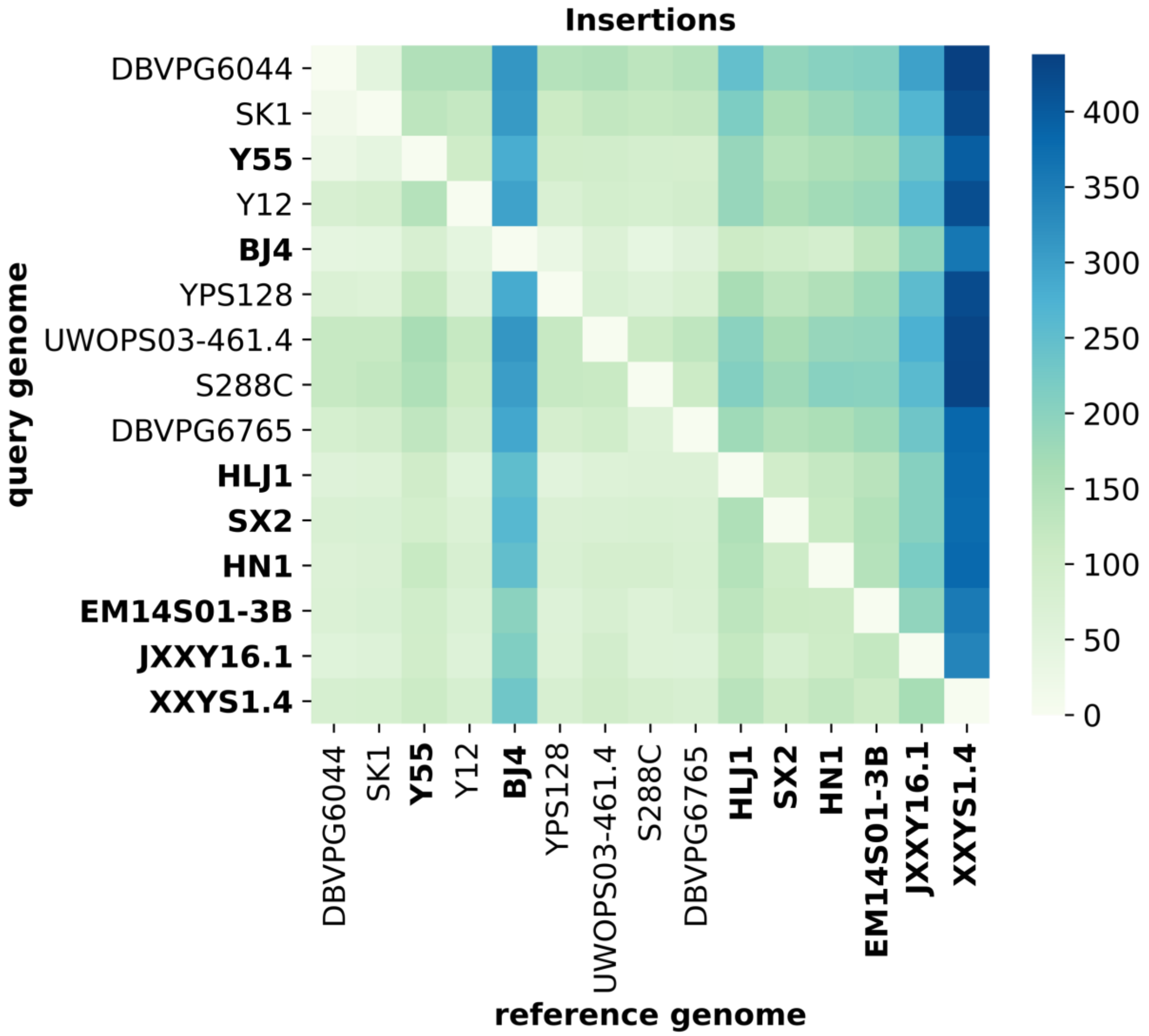

Figure S15: Insertions within *Saccharomyces cerevisiae*. Pairwise comparisons among all *Saccharomyces cerevisiae* genome assemblies depicting the total number of variations. Order of genome assemblies is consistent with the species tree (**Fig. 1**). New long-read genome assemblies presented in this study are indicated in bold.

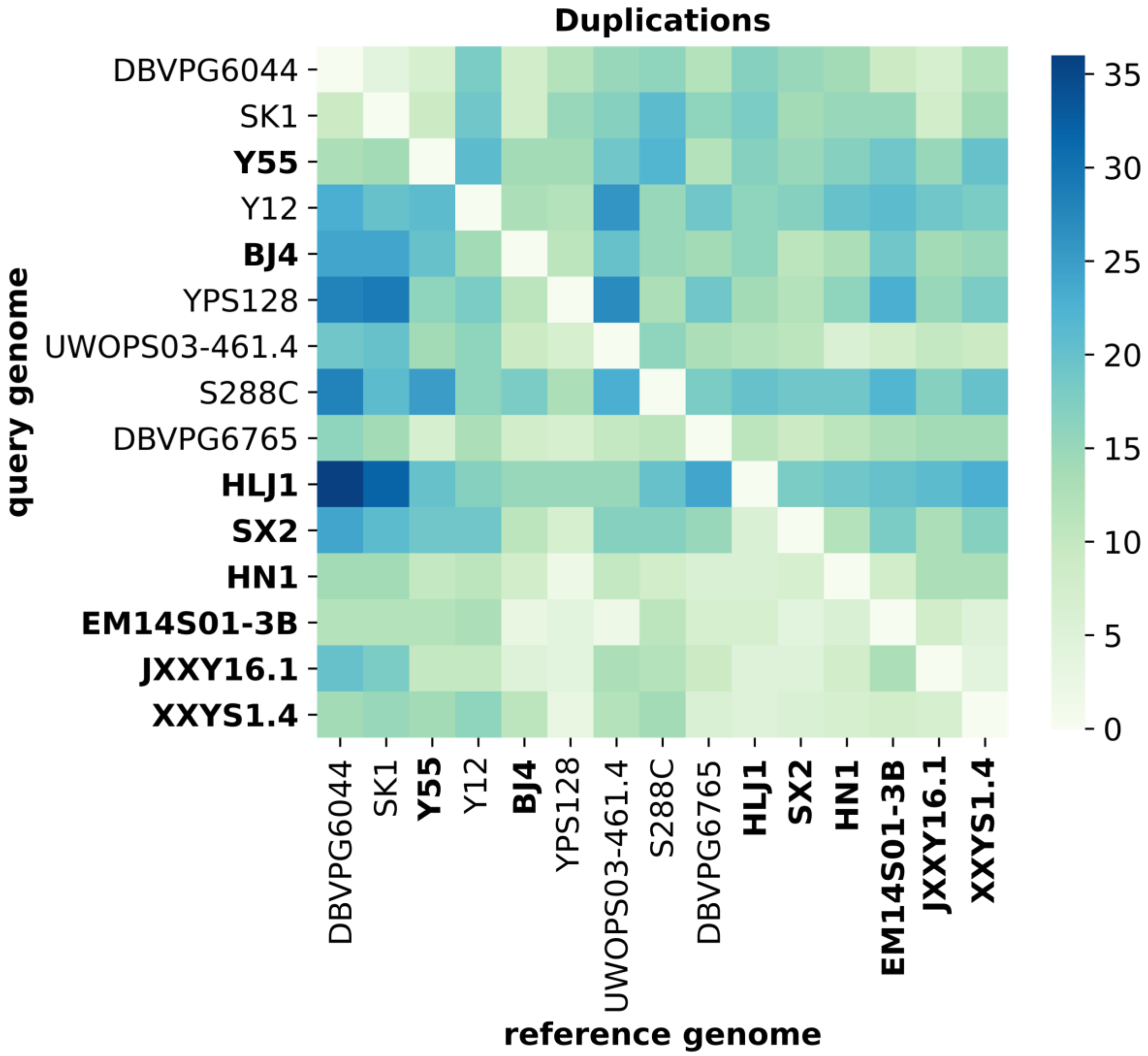

Figure S16: Duplications within *Saccharomyces cerevisiae*. Pairwise comparisons among all *Saccharomyces cerevisiae* genome assemblies depicting the total number of variations. Order of genome assemblies is consistent with the species tree (**Fig. 1**). New long-read genome assemblies presented in this study are indicated in bold.

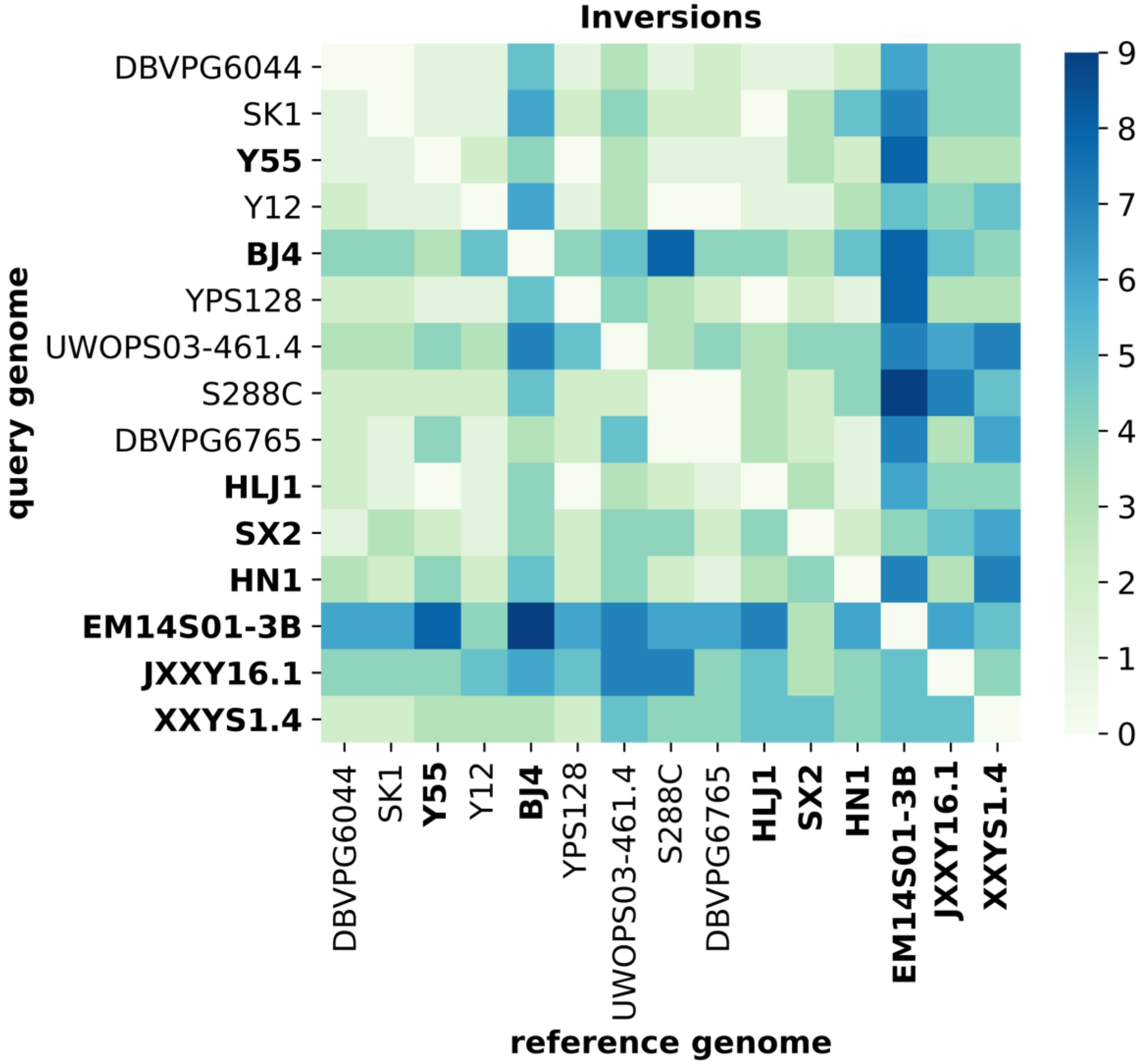

Figure S17: Inversions within *Saccharomyces cerevisiae*. Pairwise comparisons among all *Saccharomyces cerevisiae* genome assemblies depicting the total number of variations. Order of genome assemblies is consistent with the species tree (**Fig. 1**). New long-read genome assemblies presented in this study are indicated in bold.

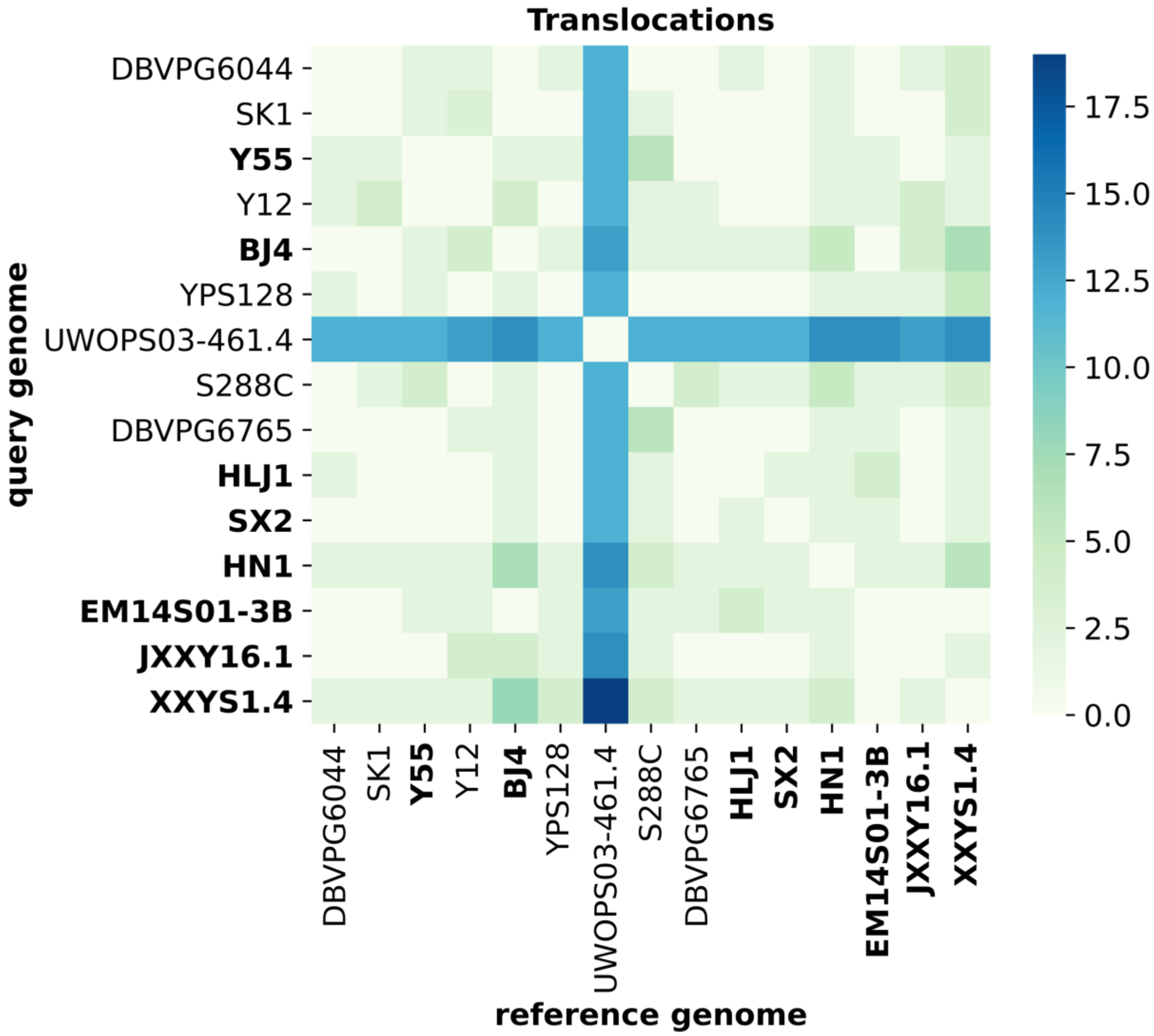

Figure S18: Translocations within *Saccharomyces cerevisiae*. Pairwise comparisons among all *Saccharomyces cerevisiae* genome assemblies depicting the total number of variations. Order of genome assemblies is consistent with the species tree (**Fig. 1**). New long-read genome assemblies presented in this study are indicated in bold.

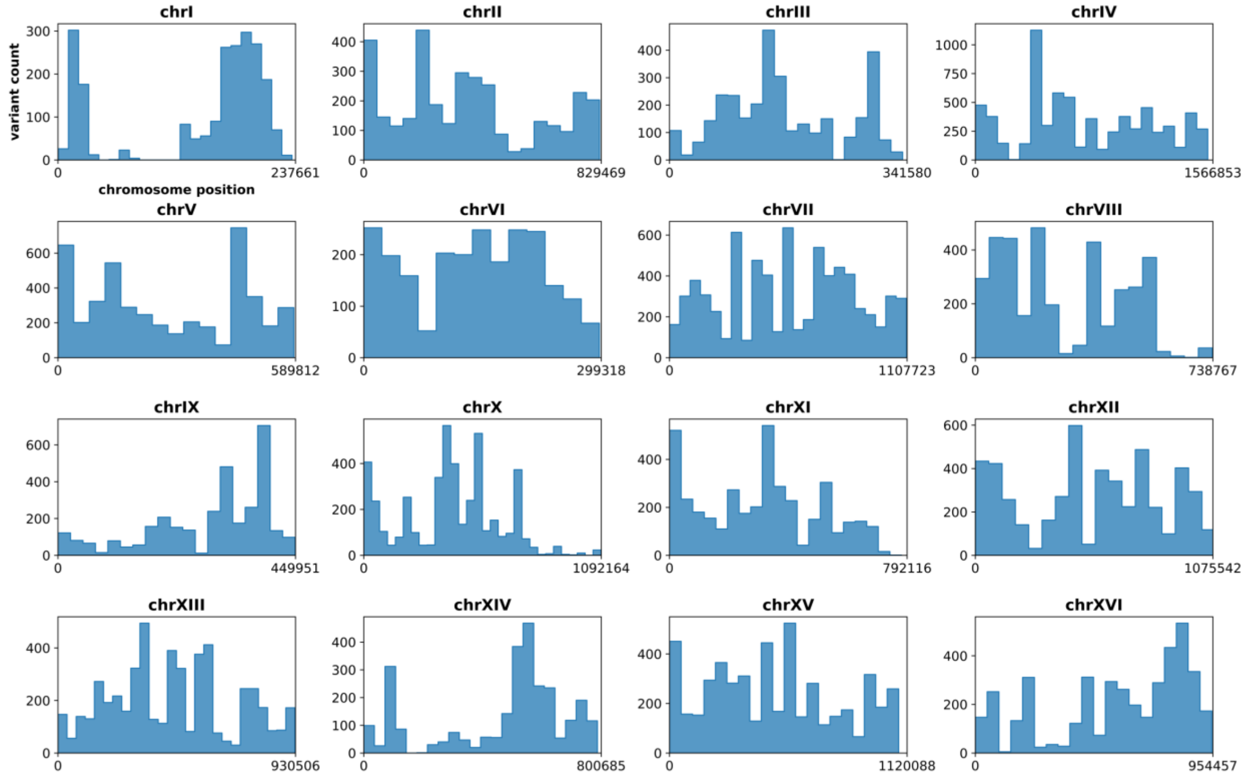

Figure S19: Distribution of structural variations along chromosomes. Histograms depicting the distribution of structural variations for each chromosome. Structural variations from all pairwise comparisons were included.

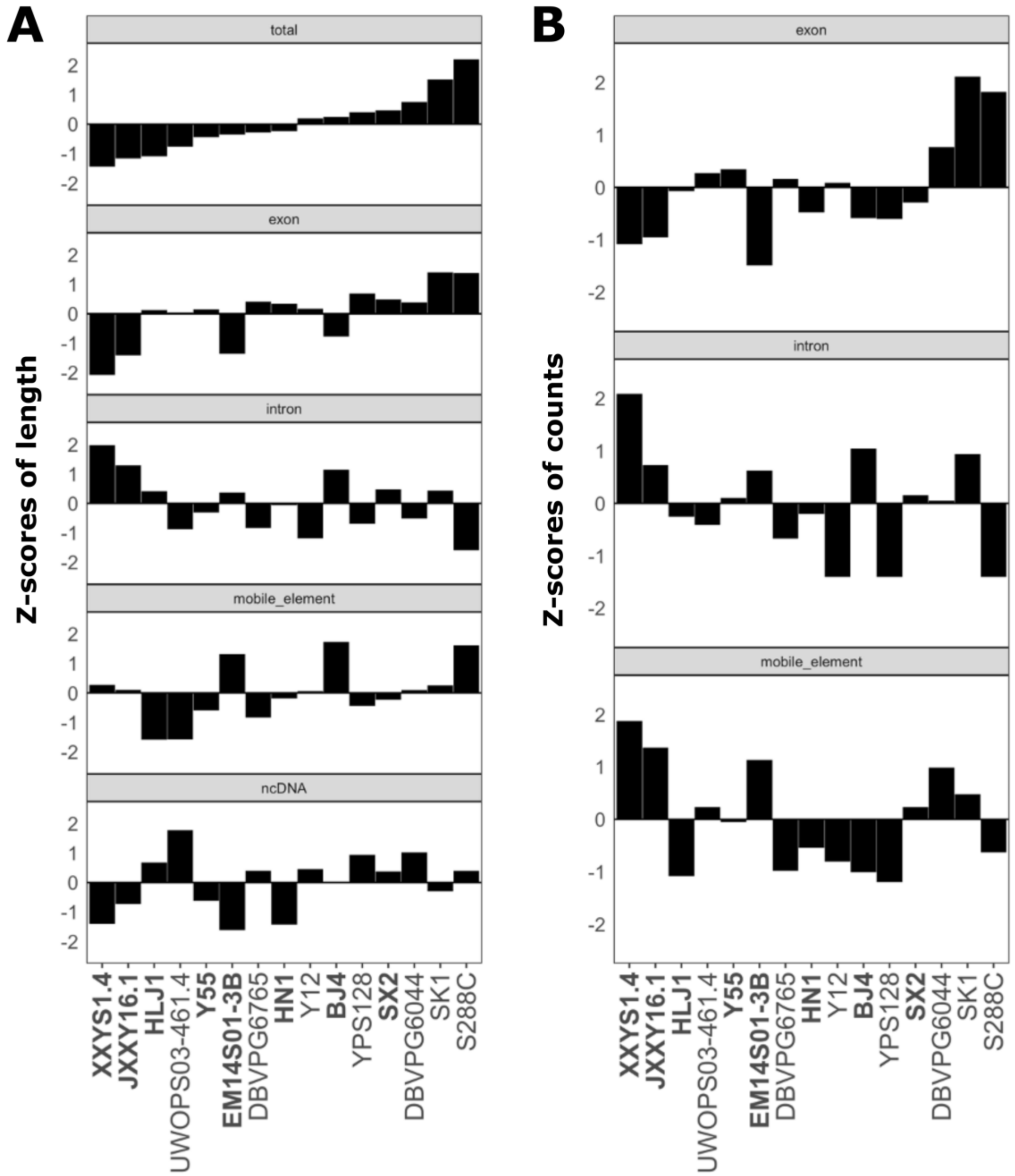

Figure S20: Nuclear genome content (A) Composite lengths in base pairs of genomic features relative to overall feature means for *S. cerevisiae* for total genomic length ($\overline{x}\pm$sd = 11.9$\pm$0.1 Mbp), exon regions ($\overline{x}\pm$sd = 8.4$\pm$0.8 Mbp), intron regions ($\overline{x}\pm$sd = 79.3$\pm$5.3 kbp), and transposable elements (TEs) ($\overline{x}\pm$sd = 218.6$\pm$84.9 kbp). **(B)** Total counts of genomic features relative to overall feature means for *S. cerevisiae* for exon regions ($\overline{x}\pm$sd = 6104.1$\pm$54.3), intron regions ($\overline{x}\pm$sd = 79.9$\pm$19.2), and TEs ($\overline{x}\pm$sd = 249.7$\pm$68.9). Strains are ordered according to the total genomic length. Strains sequenced for this study are in bold.

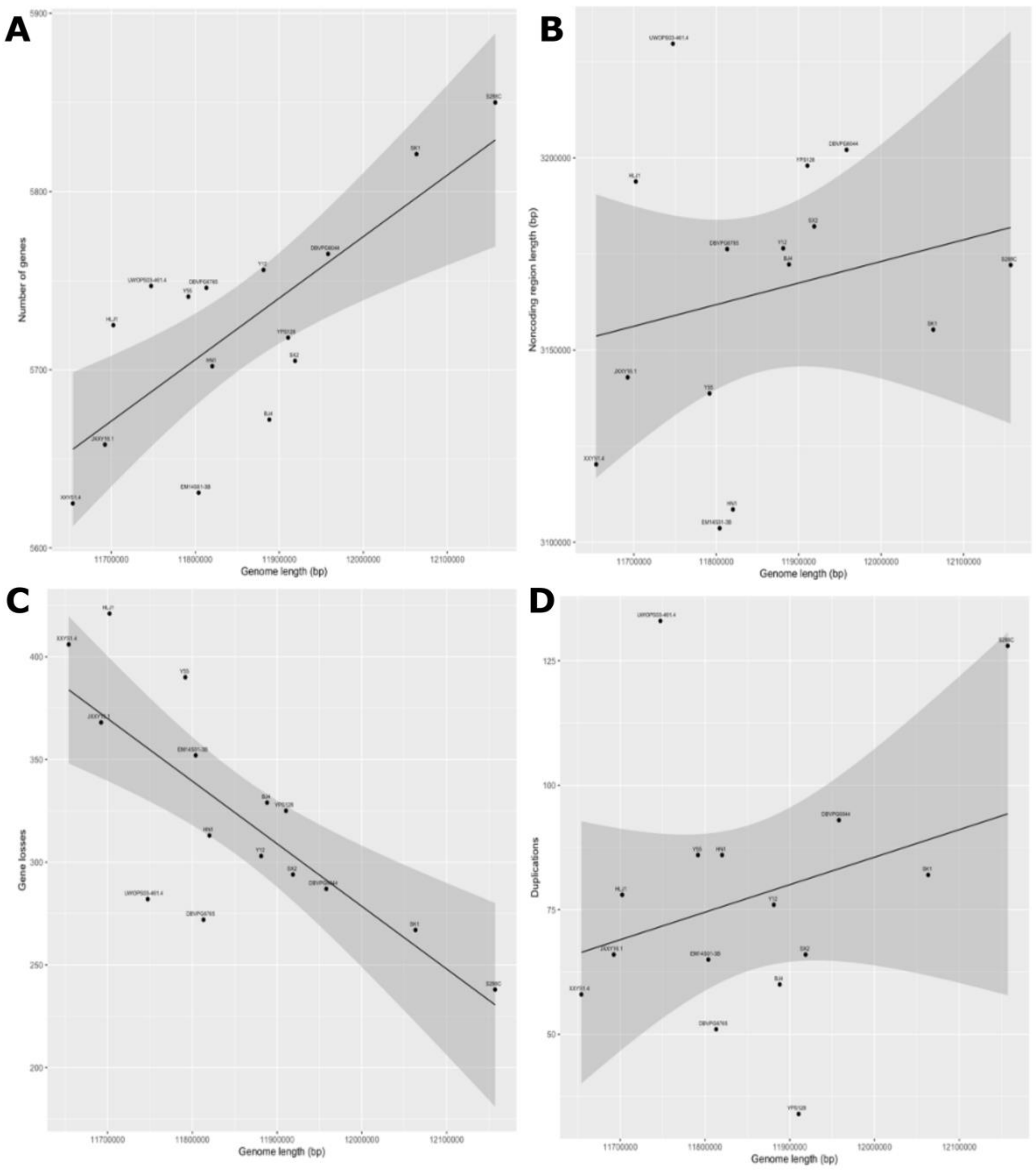

Figure S21. Nuclear content and genome size correlation in *S. cerevisiae*. Genome size and major components of genome content show (**A**) a significant positive correlation between gene number and genome size ($R^2 = 0.534$; p = 0.001) and (**B**) no correlation with cumulative length of non-coding regions ($R^2 = -0.027$; p = 0.444). This gene number and genome length correlation is tied to (**C**) gene losses ($R^2 = 0.568$; p = 0.001) rather than (**D**) gene duplications ($R^2 = 0.0128$; p = 0.297).

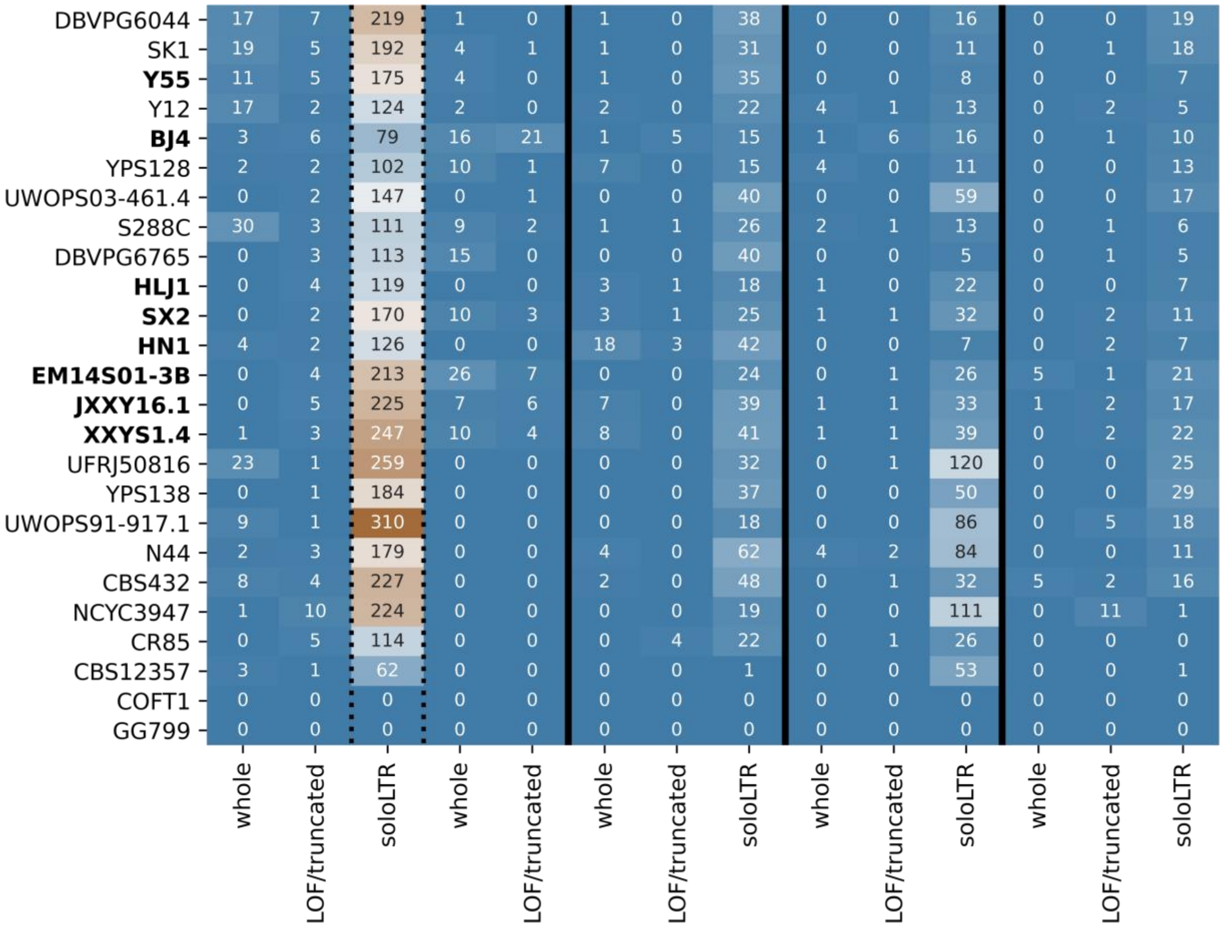

Figure S22: Annotated transposable element composition in Saccharomyces Total length of each type of transposable element found within each strain. Directly corresponds to the counts shown in **Fig. 3A**.

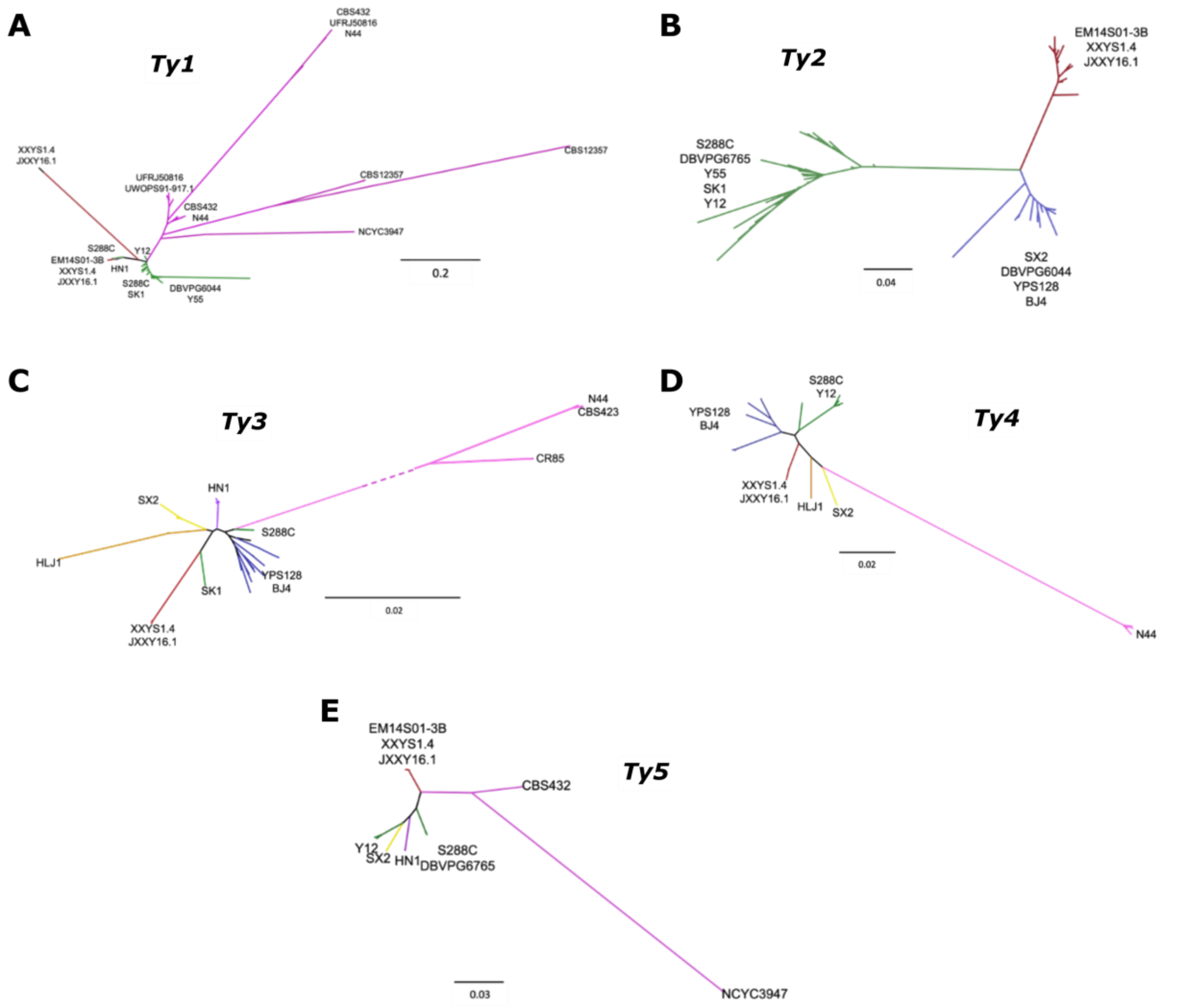

Figure S23: Gene trees of *Ty* elements. Maximum likelihood trees showing relationships between identified whole elements of **(A)** *Ty1*, **(B)** *Ty2*, **(C)** *Ty3*, **(D)** *Ty4* and **(E)** *Ty5*. Branches are colored according to rough groupings of strains with magenta indicating non-*S. cerevisiae* strains. Tips of branches are individual *Ty* elements labeled generally by the strain in which they were identified.

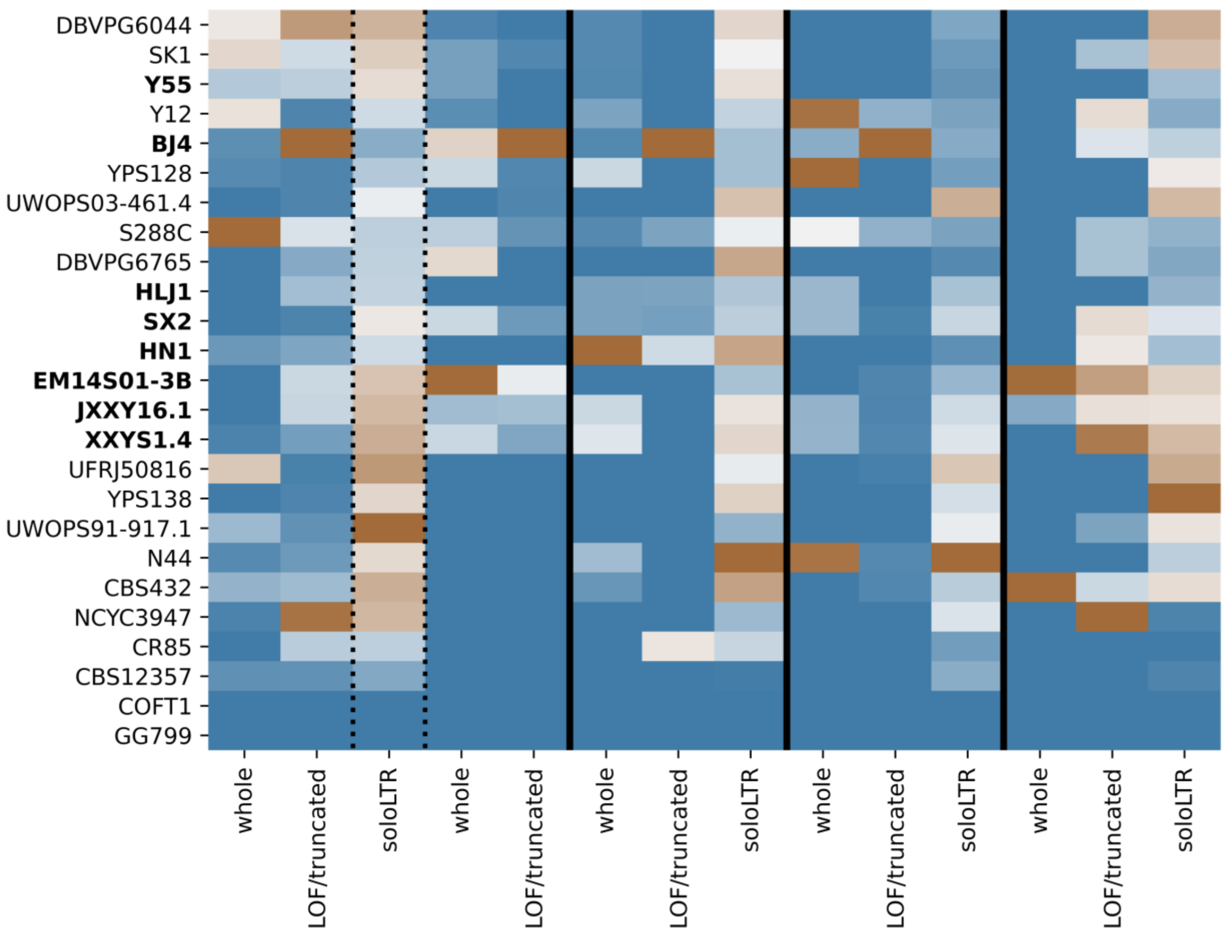

Figure S24: Length of transposable elements in Saccharomyces Total length of each type of transposable element found within each strain. Directly corresponds to the counts shown in **Fig. 3A**.

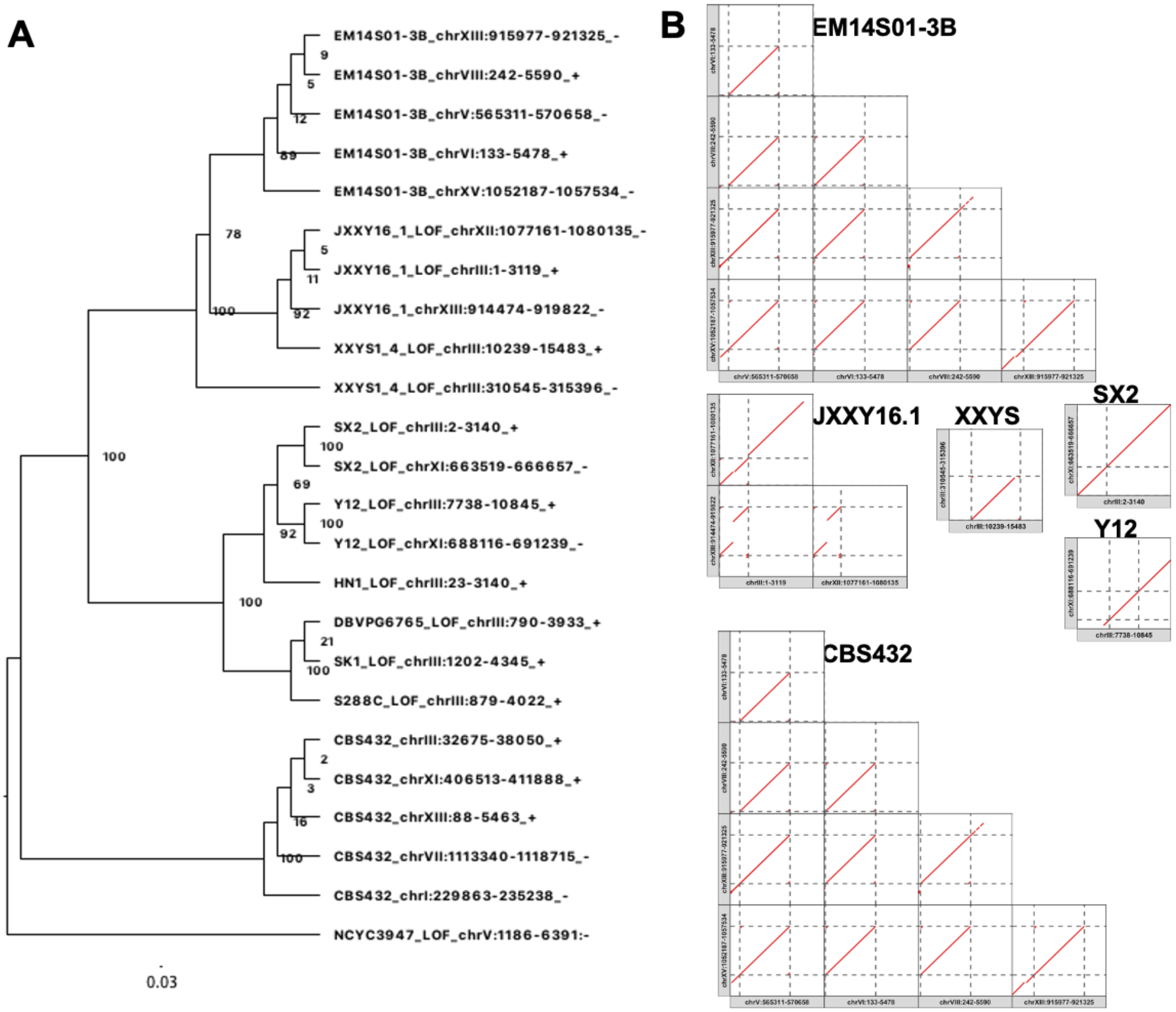

Figure S25: *Ty5* element phylogeny and dotplots. (A) Phylogenetic tree of *Ty5* elements. **(B)** Dot plots of *Ty5* elements. Dot plots shown alignments between =10kb regions within a strain containing different whole *Ty5* elements (dashed lines show the boundaries).

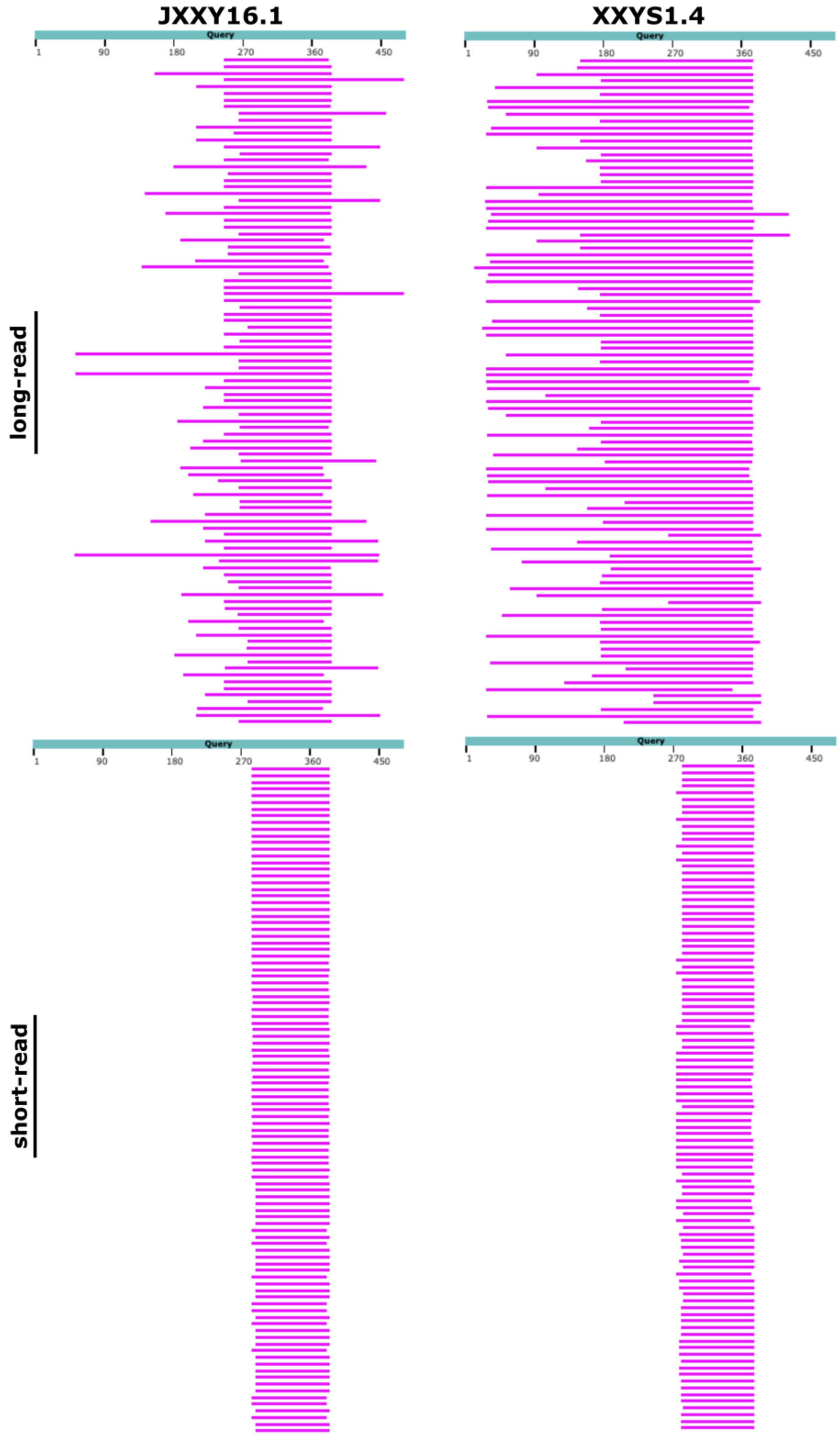

Figure S26. RPM1 BLAST analysis of short and long-read sequencing datasets. The genome sequence of RPM1 from strain S288C (483 bp) was aligned to the raw sequencing reads of short-read (Duan et al. 2018) and long-read (this study) using BLASTn (Zhang et al. 2000). Pink indicates an alignment score between 80 and 200. The top 100 aligned sequences are displayed.

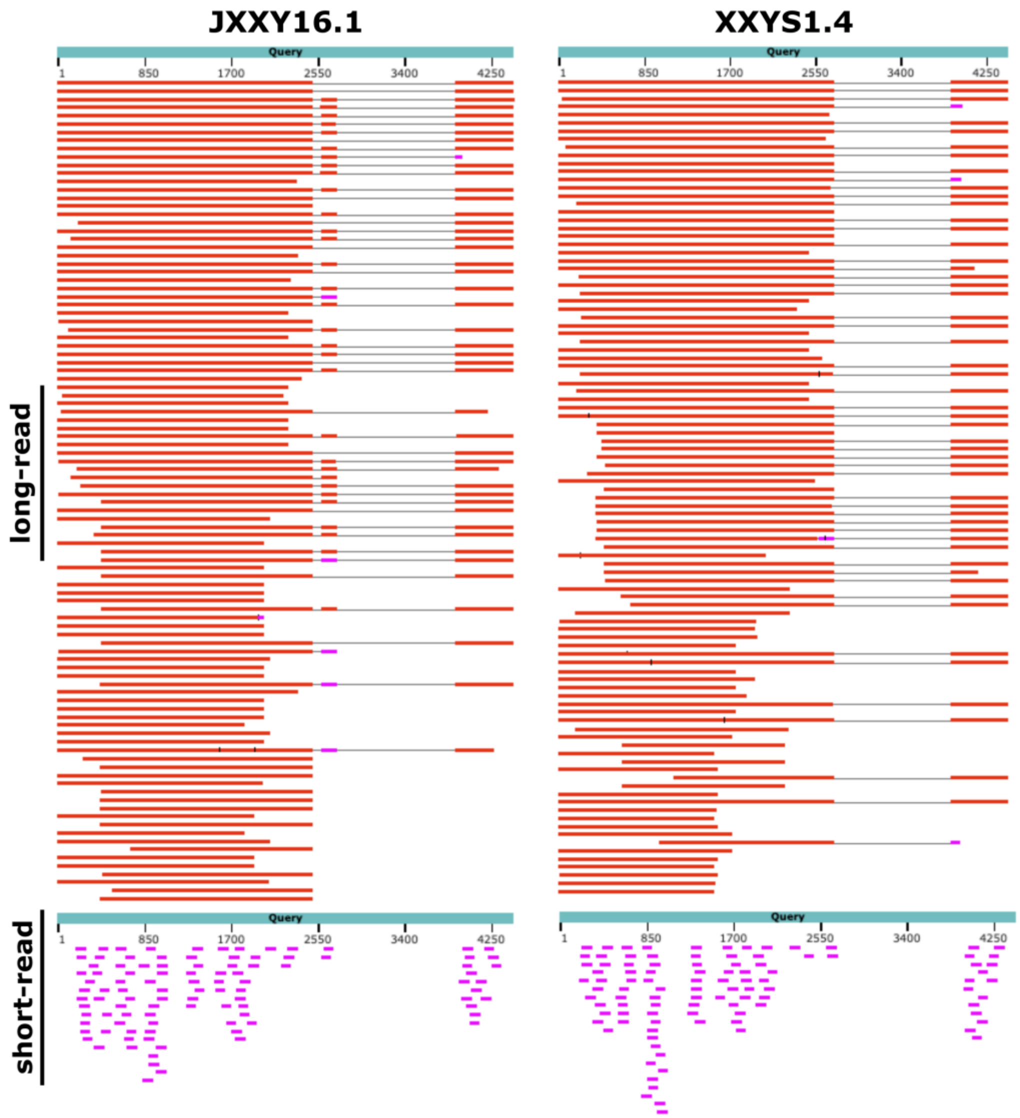

Figure S27. 21s rRNA BLAST analysis of short and long-read sequencing datasets. The genome sequence of 21s rRNA from strain S288C (4,439 bp) was aligned to the raw sequencing reads of short-read (Duan et al. 2018) and long-read (this study) using BLASTn (Zhang et al. 2000). Red indicates an alignment score >200. Pink indicates an alignment score between 80 and 200. The top 100 aligned sequences are displayed. The gapped region in the read alignments indicates the 708 bp SCEI endonuclease.

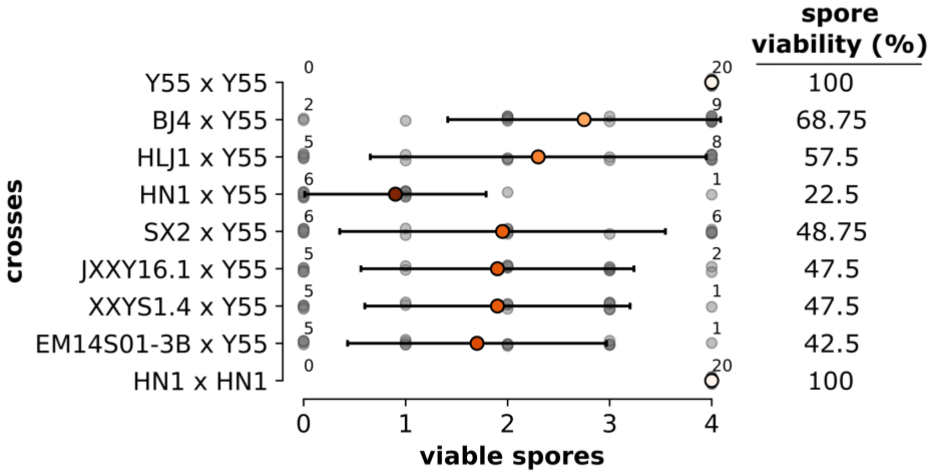

Figure S28: Spore viability of intraspecific crosses. The seven East Asian strains were mated with Y55, sporulated and the resulting tetrads dissected. For a positive control and reference Y55 was also mated with itself. 20 tetrads for cross were dissected and the number of viable spores is indicated by the grey dots. The total number of tetrads with 0 or 4 viable spores is indicated for each strain. Colored dots indicate the mean and standard deviation and are colored on a relative scale. Spore viability is calculated as the percentage of viable spores dissected from the 20 tetrads.
